# Supplementary material for: Correlations Between Body Composition and Aerobic Fitness in Elite Female Youth Water Polo Players
Source: Sports (Basel). 2025 Feb 10;13(2):51. doi: 10.3390/sports13020051 (PMC11861686; doi:10.3390/sports13020051)
Supplement: Supplementary file 1 [file sports-13-00051-s001.zip › sports-3358651-supplementary.pptx]

## Slide 1
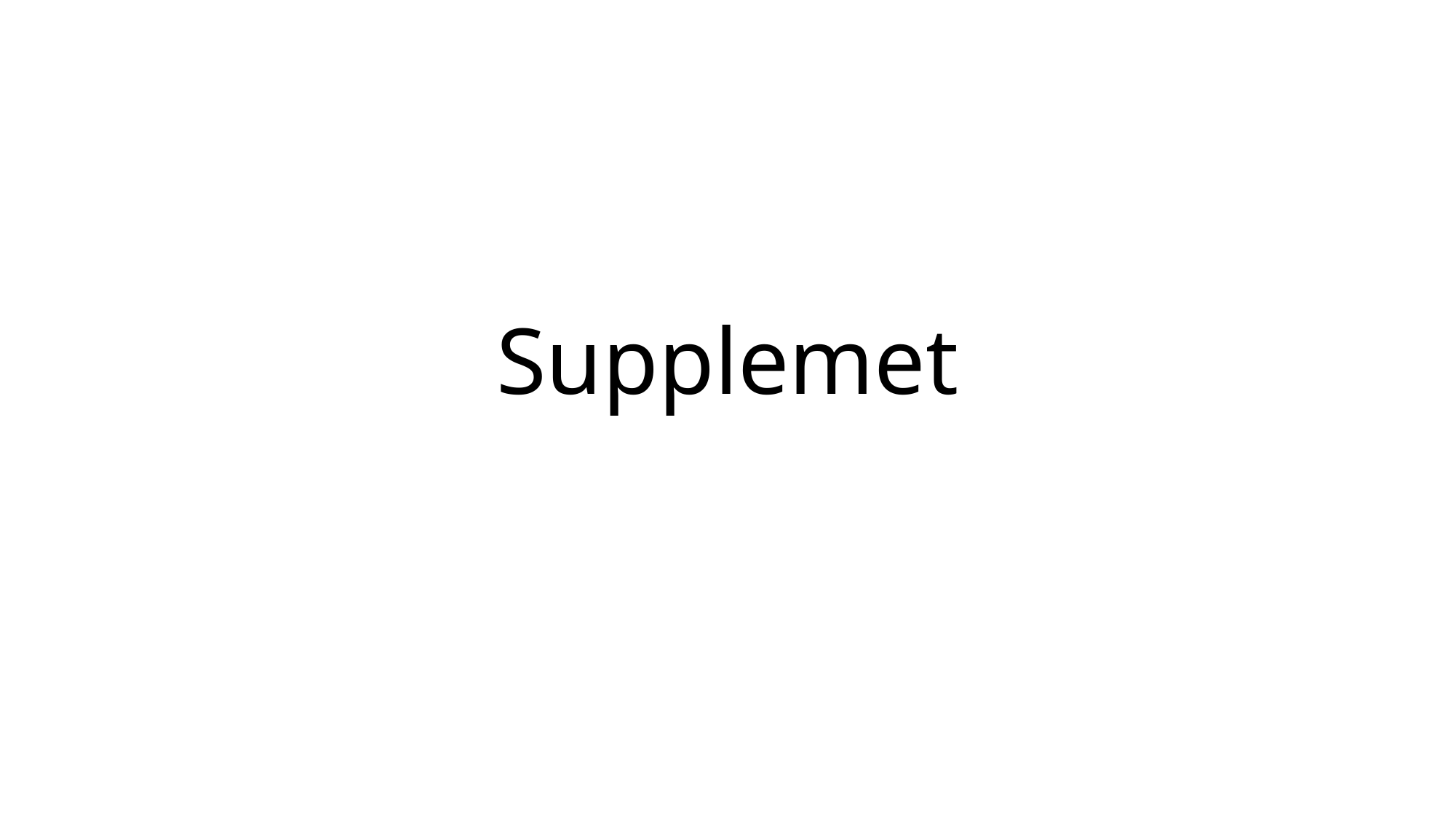

# Supplemet

## Slide 2
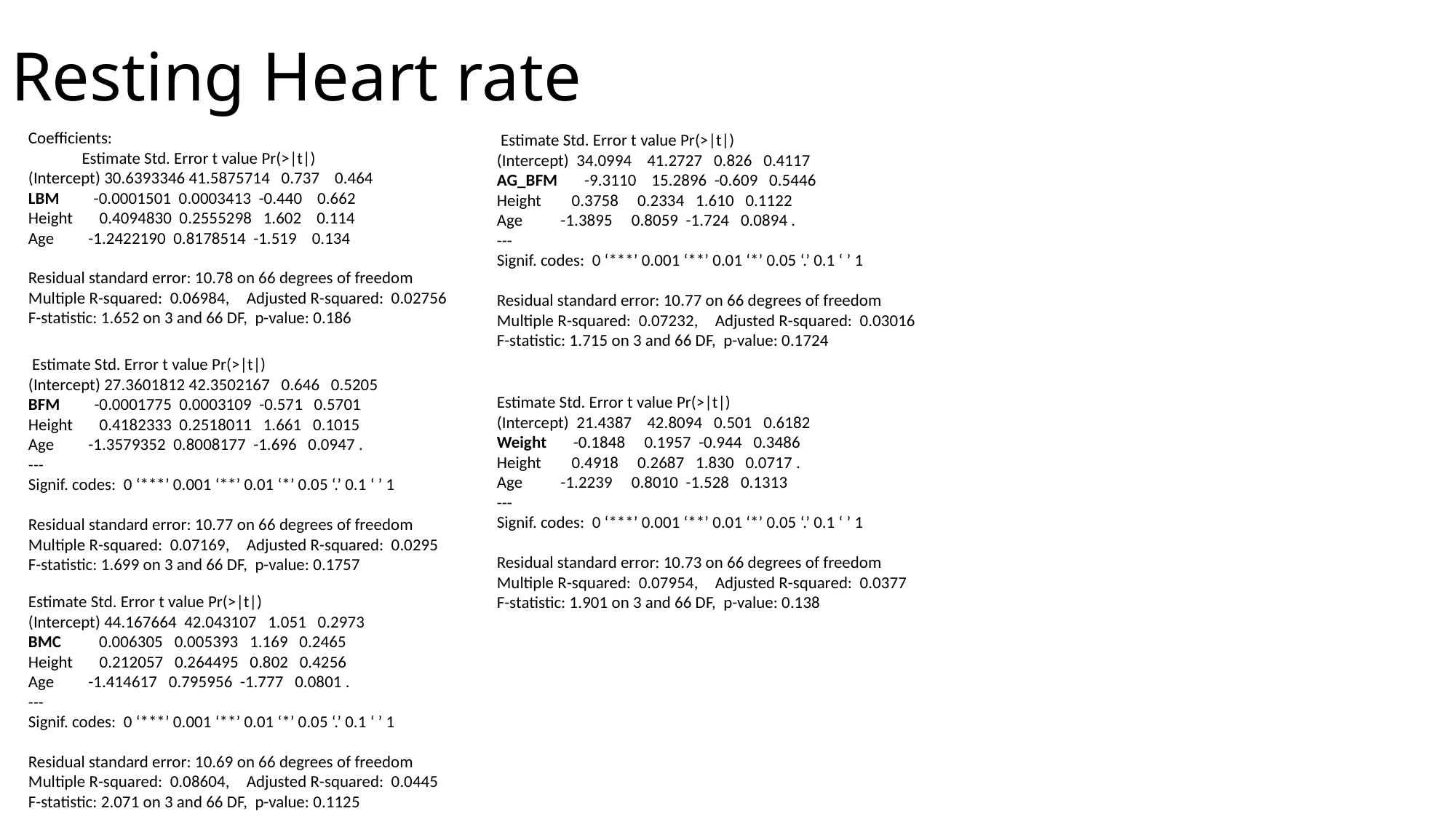

# Resting Heart rate
Coefficients:
 Estimate Std. Error t value Pr(>|t|)
(Intercept) 30.6393346 41.5875714 0.737 0.464
LBM -0.0001501 0.0003413 -0.440 0.662
Height 0.4094830 0.2555298 1.602 0.114
Age -1.2422190 0.8178514 -1.519 0.134
Residual standard error: 10.78 on 66 degrees of freedom
Multiple R-squared: 0.06984,	Adjusted R-squared: 0.02756
F-statistic: 1.652 on 3 and 66 DF, p-value: 0.186
 Estimate Std. Error t value Pr(>|t|)
(Intercept) 34.0994 41.2727 0.826 0.4117
AG_BFM -9.3110 15.2896 -0.609 0.5446
Height 0.3758 0.2334 1.610 0.1122
Age -1.3895 0.8059 -1.724 0.0894 .
---
Signif. codes: 0 ‘***’ 0.001 ‘**’ 0.01 ‘*’ 0.05 ‘.’ 0.1 ‘ ’ 1
Residual standard error: 10.77 on 66 degrees of freedom
Multiple R-squared: 0.07232,	Adjusted R-squared: 0.03016
F-statistic: 1.715 on 3 and 66 DF, p-value: 0.1724
 Estimate Std. Error t value Pr(>|t|)
(Intercept) 27.3601812 42.3502167 0.646 0.5205
BFM -0.0001775 0.0003109 -0.571 0.5701
Height 0.4182333 0.2518011 1.661 0.1015
Age -1.3579352 0.8008177 -1.696 0.0947 .
---
Signif. codes: 0 ‘***’ 0.001 ‘**’ 0.01 ‘*’ 0.05 ‘.’ 0.1 ‘ ’ 1
Residual standard error: 10.77 on 66 degrees of freedom
Multiple R-squared: 0.07169,	Adjusted R-squared: 0.0295
F-statistic: 1.699 on 3 and 66 DF, p-value: 0.1757
Estimate Std. Error t value Pr(>|t|)
(Intercept) 21.4387 42.8094 0.501 0.6182
Weight -0.1848 0.1957 -0.944 0.3486
Height 0.4918 0.2687 1.830 0.0717 .
Age -1.2239 0.8010 -1.528 0.1313
---
Signif. codes: 0 ‘***’ 0.001 ‘**’ 0.01 ‘*’ 0.05 ‘.’ 0.1 ‘ ’ 1
Residual standard error: 10.73 on 66 degrees of freedom
Multiple R-squared: 0.07954,	Adjusted R-squared: 0.0377
F-statistic: 1.901 on 3 and 66 DF, p-value: 0.138
Estimate Std. Error t value Pr(>|t|)
(Intercept) 44.167664 42.043107 1.051 0.2973
BMC 0.006305 0.005393 1.169 0.2465
Height 0.212057 0.264495 0.802 0.4256
Age -1.414617 0.795956 -1.777 0.0801 .
---
Signif. codes: 0 ‘***’ 0.001 ‘**’ 0.01 ‘*’ 0.05 ‘.’ 0.1 ‘ ’ 1
Residual standard error: 10.69 on 66 degrees of freedom
Multiple R-squared: 0.08604,	Adjusted R-squared: 0.0445
F-statistic: 2.071 on 3 and 66 DF, p-value: 0.1125

## Slide 3
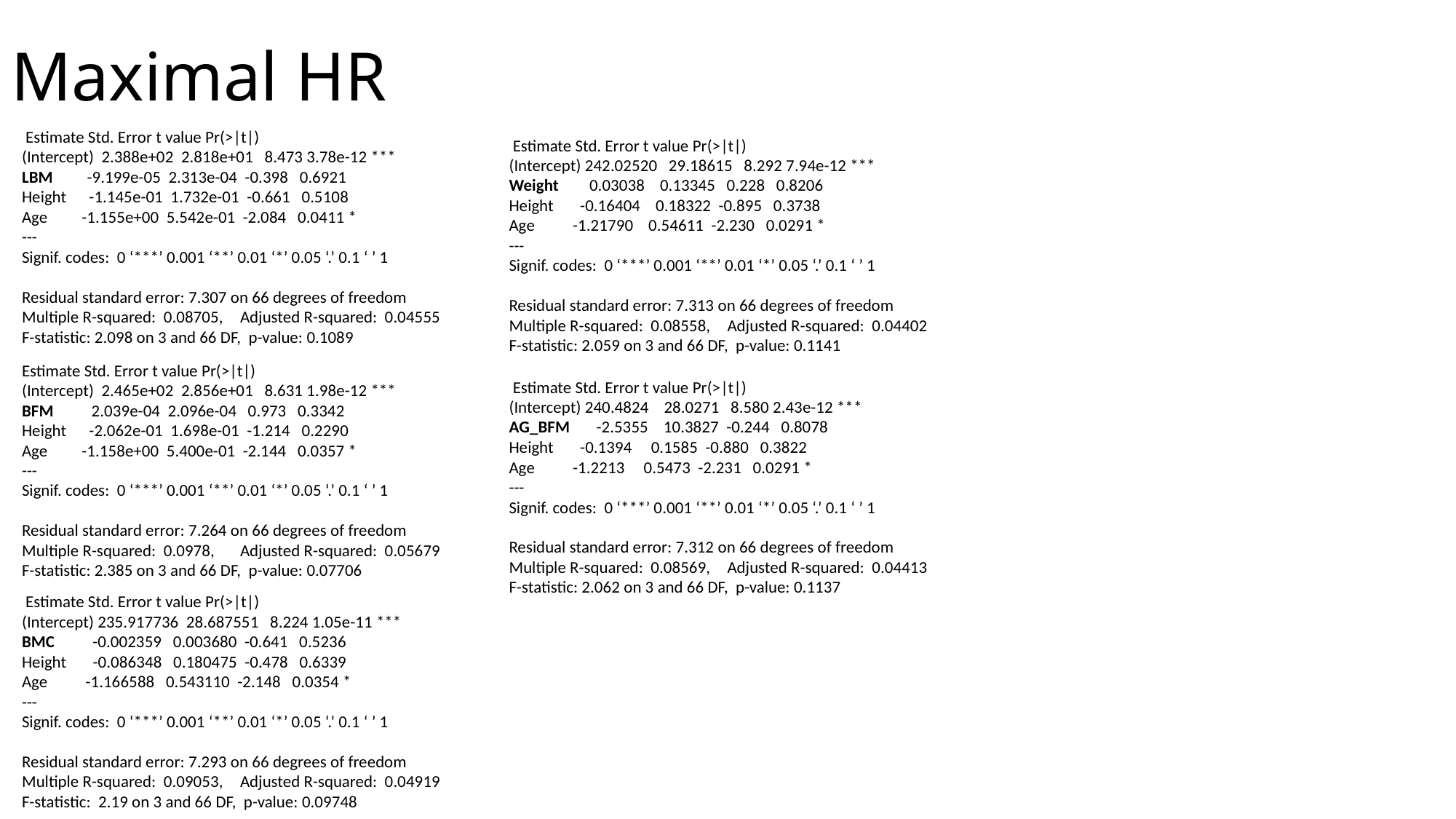

# Maximal HR
 Estimate Std. Error t value Pr(>|t|)
(Intercept) 2.388e+02 2.818e+01 8.473 3.78e-12 ***
LBM -9.199e-05 2.313e-04 -0.398 0.6921
Height -1.145e-01 1.732e-01 -0.661 0.5108
Age -1.155e+00 5.542e-01 -2.084 0.0411 *
---
Signif. codes: 0 ‘***’ 0.001 ‘**’ 0.01 ‘*’ 0.05 ‘.’ 0.1 ‘ ’ 1
Residual standard error: 7.307 on 66 degrees of freedom
Multiple R-squared: 0.08705,	Adjusted R-squared: 0.04555
F-statistic: 2.098 on 3 and 66 DF, p-value: 0.1089
 Estimate Std. Error t value Pr(>|t|)
(Intercept) 242.02520 29.18615 8.292 7.94e-12 ***
Weight 0.03038 0.13345 0.228 0.8206
Height -0.16404 0.18322 -0.895 0.3738
Age -1.21790 0.54611 -2.230 0.0291 *
---
Signif. codes: 0 ‘***’ 0.001 ‘**’ 0.01 ‘*’ 0.05 ‘.’ 0.1 ‘ ’ 1
Residual standard error: 7.313 on 66 degrees of freedom
Multiple R-squared: 0.08558,	Adjusted R-squared: 0.04402
F-statistic: 2.059 on 3 and 66 DF, p-value: 0.1141
Estimate Std. Error t value Pr(>|t|)
(Intercept) 2.465e+02 2.856e+01 8.631 1.98e-12 ***
BFM 2.039e-04 2.096e-04 0.973 0.3342
Height -2.062e-01 1.698e-01 -1.214 0.2290
Age -1.158e+00 5.400e-01 -2.144 0.0357 *
---
Signif. codes: 0 ‘***’ 0.001 ‘**’ 0.01 ‘*’ 0.05 ‘.’ 0.1 ‘ ’ 1
Residual standard error: 7.264 on 66 degrees of freedom
Multiple R-squared: 0.0978,	Adjusted R-squared: 0.05679
F-statistic: 2.385 on 3 and 66 DF, p-value: 0.07706
 Estimate Std. Error t value Pr(>|t|)
(Intercept) 240.4824 28.0271 8.580 2.43e-12 ***
AG_BFM -2.5355 10.3827 -0.244 0.8078
Height -0.1394 0.1585 -0.880 0.3822
Age -1.2213 0.5473 -2.231 0.0291 *
---
Signif. codes: 0 ‘***’ 0.001 ‘**’ 0.01 ‘*’ 0.05 ‘.’ 0.1 ‘ ’ 1
Residual standard error: 7.312 on 66 degrees of freedom
Multiple R-squared: 0.08569,	Adjusted R-squared: 0.04413
F-statistic: 2.062 on 3 and 66 DF, p-value: 0.1137
 Estimate Std. Error t value Pr(>|t|)
(Intercept) 235.917736 28.687551 8.224 1.05e-11 ***
BMC -0.002359 0.003680 -0.641 0.5236
Height -0.086348 0.180475 -0.478 0.6339
Age -1.166588 0.543110 -2.148 0.0354 *
---
Signif. codes: 0 ‘***’ 0.001 ‘**’ 0.01 ‘*’ 0.05 ‘.’ 0.1 ‘ ’ 1
Residual standard error: 7.293 on 66 degrees of freedom
Multiple R-squared: 0.09053,	Adjusted R-squared: 0.04919
F-statistic: 2.19 on 3 and 66 DF, p-value: 0.09748

## Slide 4
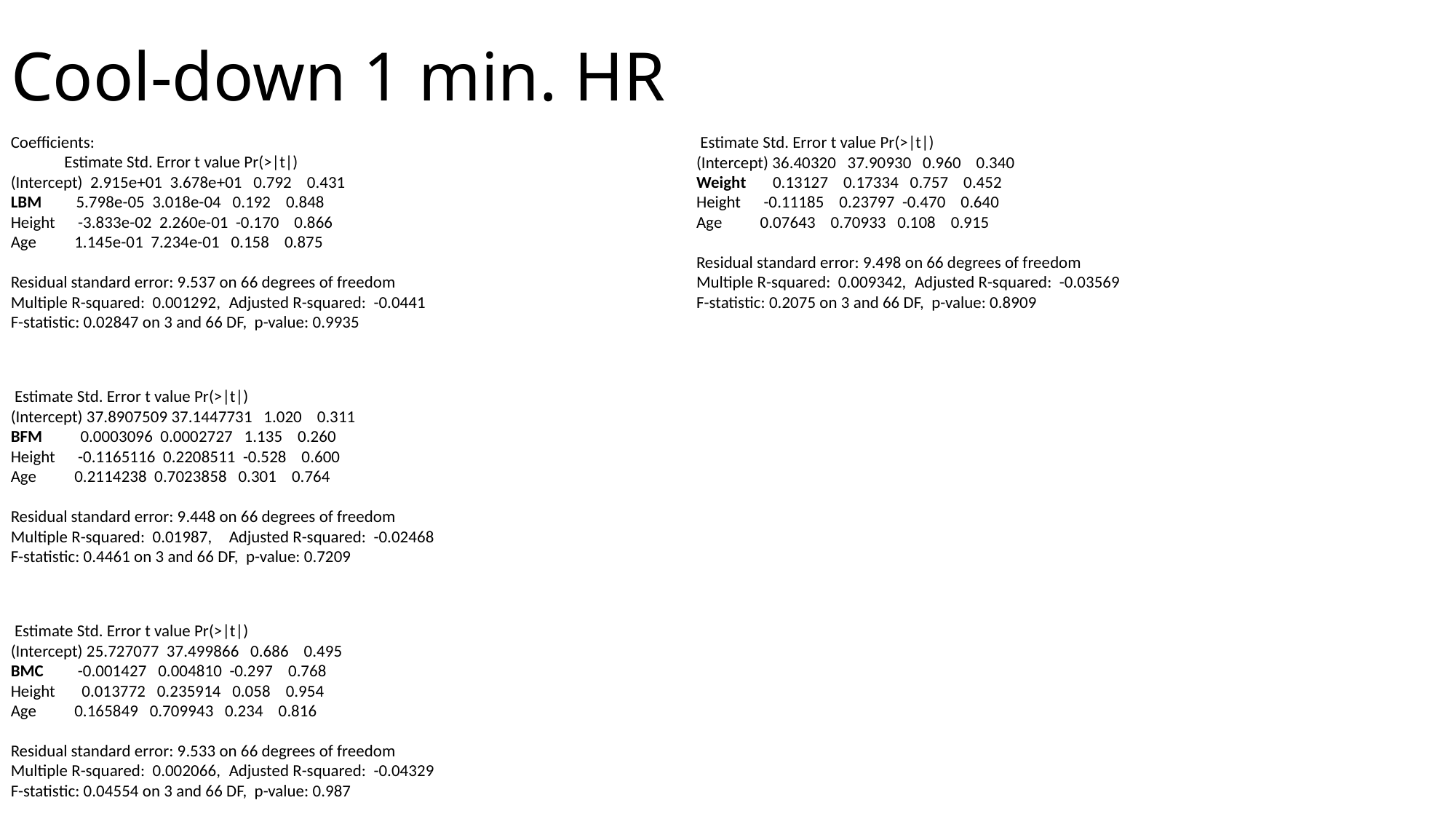

# Cool-down 1 min. HR
Coefficients:
 Estimate Std. Error t value Pr(>|t|)
(Intercept) 2.915e+01 3.678e+01 0.792 0.431
LBM 5.798e-05 3.018e-04 0.192 0.848
Height -3.833e-02 2.260e-01 -0.170 0.866
Age 1.145e-01 7.234e-01 0.158 0.875
Residual standard error: 9.537 on 66 degrees of freedom
Multiple R-squared: 0.001292,	Adjusted R-squared: -0.0441
F-statistic: 0.02847 on 3 and 66 DF, p-value: 0.9935
 Estimate Std. Error t value Pr(>|t|)
(Intercept) 36.40320 37.90930 0.960 0.340
Weight 0.13127 0.17334 0.757 0.452
Height -0.11185 0.23797 -0.470 0.640
Age 0.07643 0.70933 0.108 0.915
Residual standard error: 9.498 on 66 degrees of freedom
Multiple R-squared: 0.009342,	Adjusted R-squared: -0.03569
F-statistic: 0.2075 on 3 and 66 DF, p-value: 0.8909
 Estimate Std. Error t value Pr(>|t|)
(Intercept) 37.8907509 37.1447731 1.020 0.311
BFM 0.0003096 0.0002727 1.135 0.260
Height -0.1165116 0.2208511 -0.528 0.600
Age 0.2114238 0.7023858 0.301 0.764
Residual standard error: 9.448 on 66 degrees of freedom
Multiple R-squared: 0.01987,	Adjusted R-squared: -0.02468
F-statistic: 0.4461 on 3 and 66 DF, p-value: 0.7209
 Estimate Std. Error t value Pr(>|t|)
(Intercept) 25.727077 37.499866 0.686 0.495
BMC -0.001427 0.004810 -0.297 0.768
Height 0.013772 0.235914 0.058 0.954
Age 0.165849 0.709943 0.234 0.816
Residual standard error: 9.533 on 66 degrees of freedom
Multiple R-squared: 0.002066,	Adjusted R-squared: -0.04329
F-statistic: 0.04554 on 3 and 66 DF, p-value: 0.987

## Slide 5
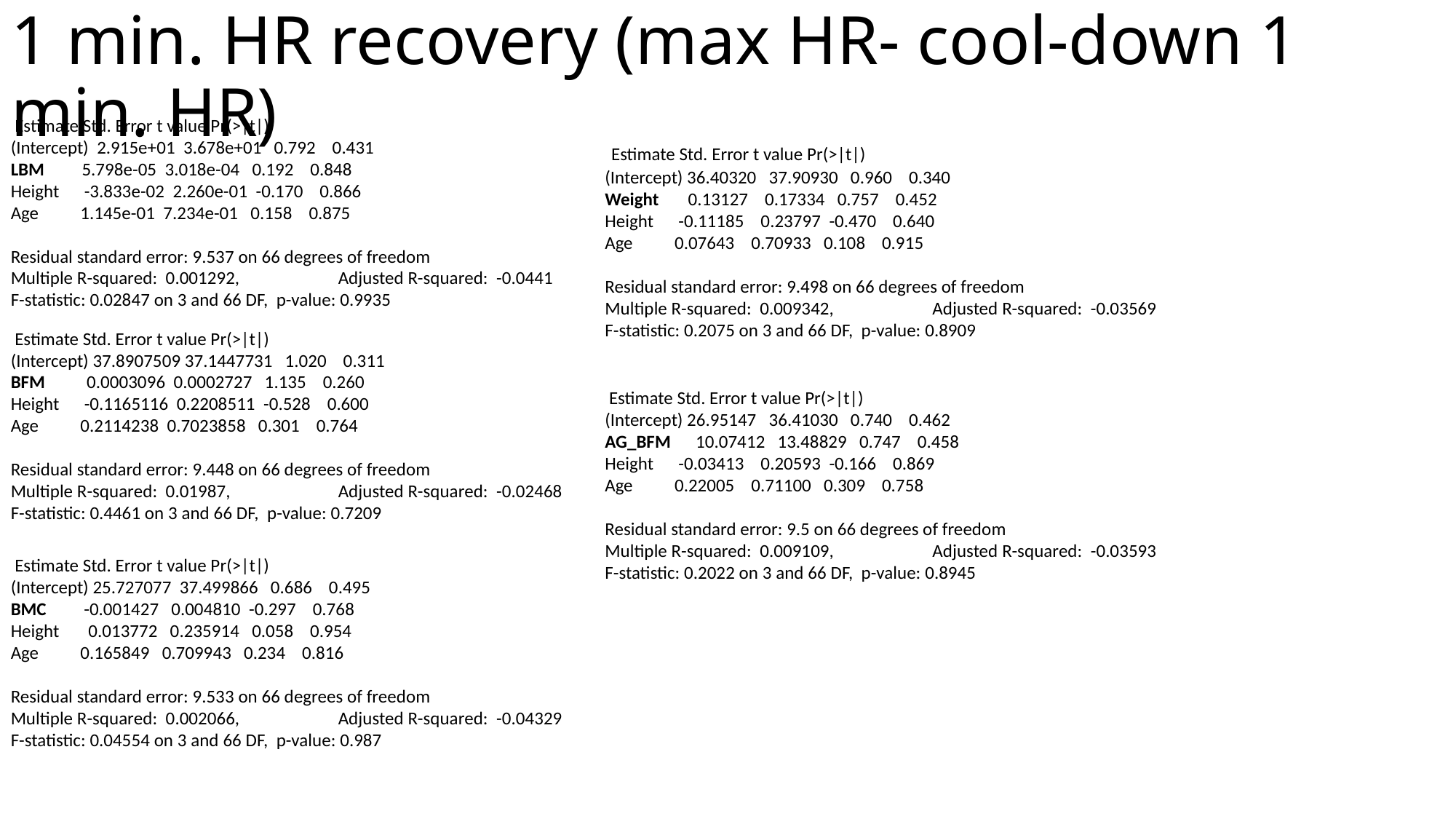

# 1 min. HR recovery (max HR- cool-down 1 min. HR)
 Estimate Std. Error t value Pr(>|t|)
(Intercept) 2.915e+01 3.678e+01 0.792 0.431
LBM 5.798e-05 3.018e-04 0.192 0.848
Height -3.833e-02 2.260e-01 -0.170 0.866
Age 1.145e-01 7.234e-01 0.158 0.875
Residual standard error: 9.537 on 66 degrees of freedom
Multiple R-squared: 0.001292,	Adjusted R-squared: -0.0441
F-statistic: 0.02847 on 3 and 66 DF, p-value: 0.9935
 Estimate Std. Error t value Pr(>|t|)
(Intercept) 36.40320 37.90930 0.960 0.340
Weight 0.13127 0.17334 0.757 0.452
Height -0.11185 0.23797 -0.470 0.640
Age 0.07643 0.70933 0.108 0.915
Residual standard error: 9.498 on 66 degrees of freedom
Multiple R-squared: 0.009342,	Adjusted R-squared: -0.03569
F-statistic: 0.2075 on 3 and 66 DF, p-value: 0.8909
 Estimate Std. Error t value Pr(>|t|)
(Intercept) 37.8907509 37.1447731 1.020 0.311
BFM 0.0003096 0.0002727 1.135 0.260
Height -0.1165116 0.2208511 -0.528 0.600
Age 0.2114238 0.7023858 0.301 0.764
Residual standard error: 9.448 on 66 degrees of freedom
Multiple R-squared: 0.01987,	Adjusted R-squared: -0.02468
F-statistic: 0.4461 on 3 and 66 DF, p-value: 0.7209
 Estimate Std. Error t value Pr(>|t|)
(Intercept) 26.95147 36.41030 0.740 0.462
AG_BFM 10.07412 13.48829 0.747 0.458
Height -0.03413 0.20593 -0.166 0.869
Age 0.22005 0.71100 0.309 0.758
Residual standard error: 9.5 on 66 degrees of freedom
Multiple R-squared: 0.009109,	Adjusted R-squared: -0.03593
F-statistic: 0.2022 on 3 and 66 DF, p-value: 0.8945
 Estimate Std. Error t value Pr(>|t|)
(Intercept) 25.727077 37.499866 0.686 0.495
BMC -0.001427 0.004810 -0.297 0.768
Height 0.013772 0.235914 0.058 0.954
Age 0.165849 0.709943 0.234 0.816
Residual standard error: 9.533 on 66 degrees of freedom
Multiple R-squared: 0.002066,	Adjusted R-squared: -0.04329
F-statistic: 0.04554 on 3 and 66 DF, p-value: 0.987

## Slide 6
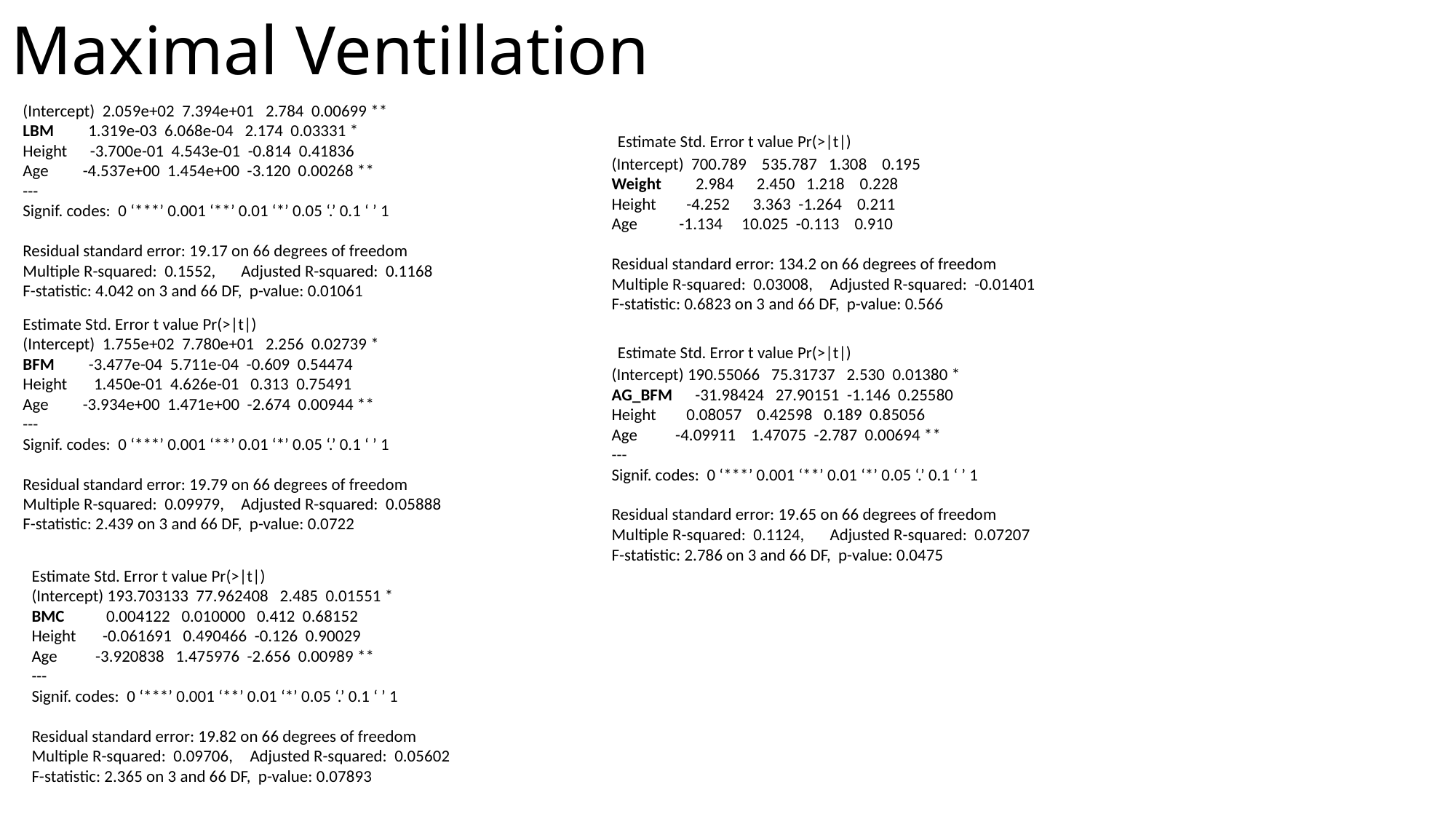

# Maximal Ventillation
(Intercept) 2.059e+02 7.394e+01 2.784 0.00699 **
LBM 1.319e-03 6.068e-04 2.174 0.03331 *
Height -3.700e-01 4.543e-01 -0.814 0.41836
Age -4.537e+00 1.454e+00 -3.120 0.00268 **
---
Signif. codes: 0 ‘***’ 0.001 ‘**’ 0.01 ‘*’ 0.05 ‘.’ 0.1 ‘ ’ 1
Residual standard error: 19.17 on 66 degrees of freedom
Multiple R-squared: 0.1552,	Adjusted R-squared: 0.1168
F-statistic: 4.042 on 3 and 66 DF, p-value: 0.01061
 Estimate Std. Error t value Pr(>|t|)
(Intercept) 700.789 535.787 1.308 0.195
Weight 2.984 2.450 1.218 0.228
Height -4.252 3.363 -1.264 0.211
Age -1.134 10.025 -0.113 0.910
Residual standard error: 134.2 on 66 degrees of freedom
Multiple R-squared: 0.03008,	Adjusted R-squared: -0.01401
F-statistic: 0.6823 on 3 and 66 DF, p-value: 0.566
Estimate Std. Error t value Pr(>|t|)
(Intercept) 1.755e+02 7.780e+01 2.256 0.02739 *
BFM -3.477e-04 5.711e-04 -0.609 0.54474
Height 1.450e-01 4.626e-01 0.313 0.75491
Age -3.934e+00 1.471e+00 -2.674 0.00944 **
---
Signif. codes: 0 ‘***’ 0.001 ‘**’ 0.01 ‘*’ 0.05 ‘.’ 0.1 ‘ ’ 1
Residual standard error: 19.79 on 66 degrees of freedom
Multiple R-squared: 0.09979,	Adjusted R-squared: 0.05888
F-statistic: 2.439 on 3 and 66 DF, p-value: 0.0722
 Estimate Std. Error t value Pr(>|t|)
(Intercept) 190.55066 75.31737 2.530 0.01380 *
AG_BFM -31.98424 27.90151 -1.146 0.25580
Height 0.08057 0.42598 0.189 0.85056
Age -4.09911 1.47075 -2.787 0.00694 **
---
Signif. codes: 0 ‘***’ 0.001 ‘**’ 0.01 ‘*’ 0.05 ‘.’ 0.1 ‘ ’ 1
Residual standard error: 19.65 on 66 degrees of freedom
Multiple R-squared: 0.1124,	Adjusted R-squared: 0.07207
F-statistic: 2.786 on 3 and 66 DF, p-value: 0.0475
Estimate Std. Error t value Pr(>|t|)
(Intercept) 193.703133 77.962408 2.485 0.01551 *
BMC 0.004122 0.010000 0.412 0.68152
Height -0.061691 0.490466 -0.126 0.90029
Age -3.920838 1.475976 -2.656 0.00989 **
---
Signif. codes: 0 ‘***’ 0.001 ‘**’ 0.01 ‘*’ 0.05 ‘.’ 0.1 ‘ ’ 1
Residual standard error: 19.82 on 66 degrees of freedom
Multiple R-squared: 0.09706,	Adjusted R-squared: 0.05602
F-statistic: 2.365 on 3 and 66 DF, p-value: 0.07893

## Slide 7
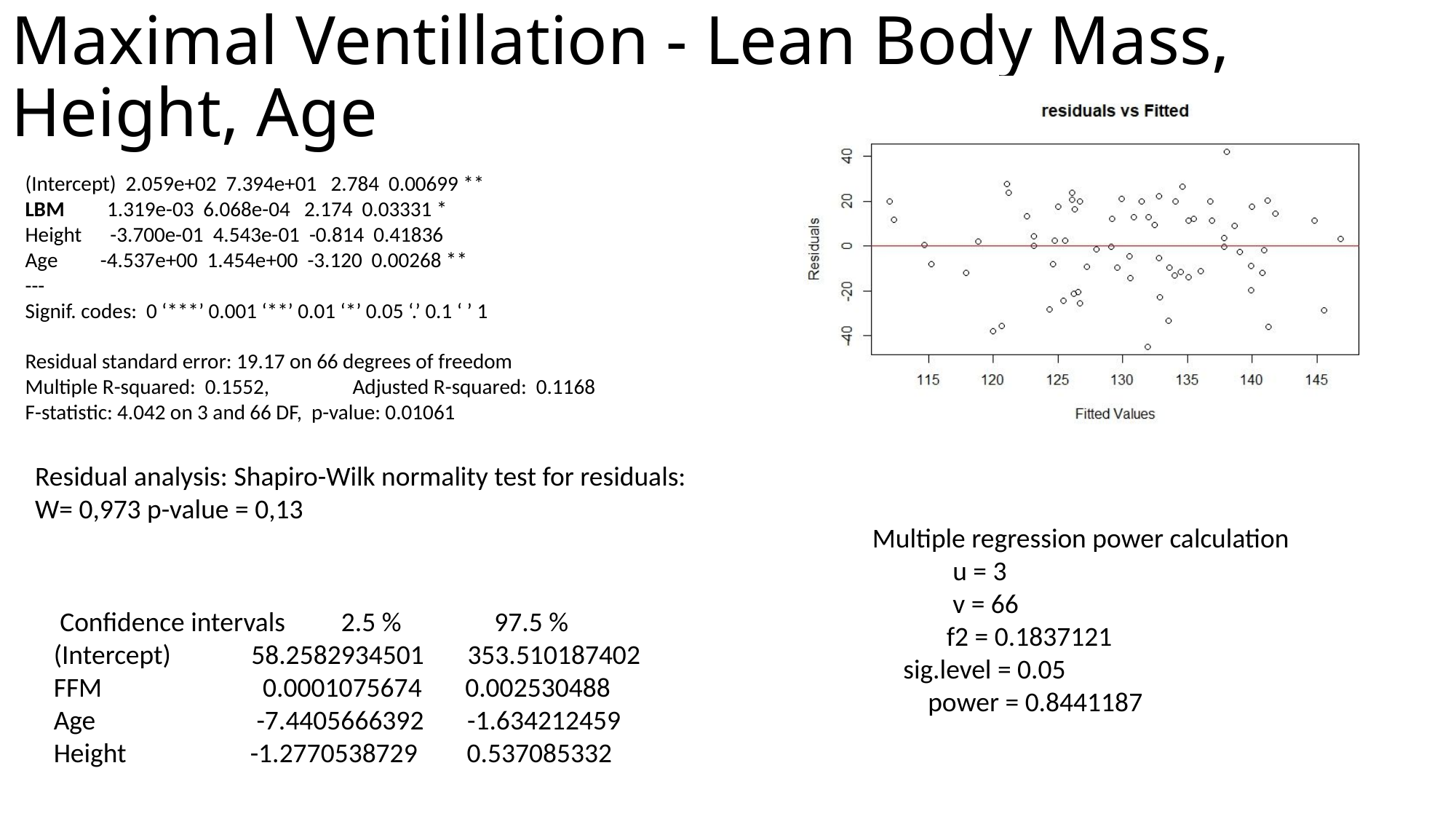

# Maximal Ventillation - Lean Body Mass, Height, Age
(Intercept) 2.059e+02 7.394e+01 2.784 0.00699 **
LBM 1.319e-03 6.068e-04 2.174 0.03331 *
Height -3.700e-01 4.543e-01 -0.814 0.41836
Age -4.537e+00 1.454e+00 -3.120 0.00268 **
---
Signif. codes: 0 ‘***’ 0.001 ‘**’ 0.01 ‘*’ 0.05 ‘.’ 0.1 ‘ ’ 1
Residual standard error: 19.17 on 66 degrees of freedom
Multiple R-squared: 0.1552,	Adjusted R-squared: 0.1168
F-statistic: 4.042 on 3 and 66 DF, p-value: 0.01061
Residual analysis: Shapiro-Wilk normality test for residuals:
W= 0,973 p-value = 0,13
 Multiple regression power calculation
 u = 3
 v = 66
 f2 = 0.1837121
 sig.level = 0.05
 power = 0.8441187
 Confidence intervals 2.5 % 97.5 %
(Intercept) 58.2582934501 353.510187402
FFM 0.0001075674 0.002530488
Age -7.4405666392 -1.634212459
Height -1.2770538729 0.537085332

## Slide 8
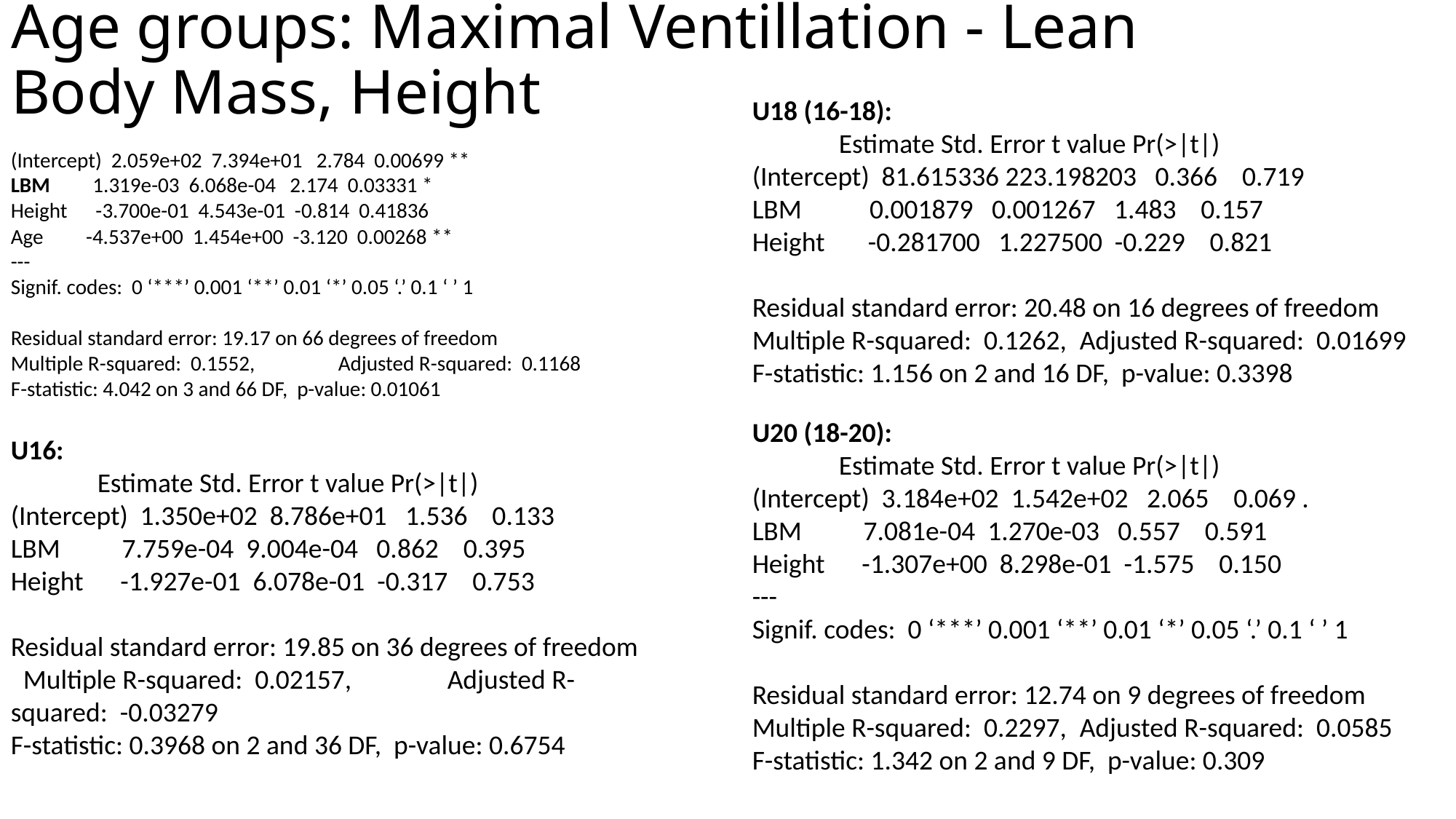

# Age groups: Maximal Ventillation - Lean Body Mass, Height
U18 (16-18):
 Estimate Std. Error t value Pr(>|t|)
(Intercept) 81.615336 223.198203 0.366 0.719
LBM 0.001879 0.001267 1.483 0.157
Height -0.281700 1.227500 -0.229 0.821
Residual standard error: 20.48 on 16 degrees of freedom
Multiple R-squared: 0.1262,	Adjusted R-squared: 0.01699
F-statistic: 1.156 on 2 and 16 DF, p-value: 0.3398
(Intercept) 2.059e+02 7.394e+01 2.784 0.00699 **
LBM 1.319e-03 6.068e-04 2.174 0.03331 *
Height -3.700e-01 4.543e-01 -0.814 0.41836
Age -4.537e+00 1.454e+00 -3.120 0.00268 **
---
Signif. codes: 0 ‘***’ 0.001 ‘**’ 0.01 ‘*’ 0.05 ‘.’ 0.1 ‘ ’ 1
Residual standard error: 19.17 on 66 degrees of freedom
Multiple R-squared: 0.1552,	Adjusted R-squared: 0.1168
F-statistic: 4.042 on 3 and 66 DF, p-value: 0.01061
U20 (18-20):
 Estimate Std. Error t value Pr(>|t|)
(Intercept) 3.184e+02 1.542e+02 2.065 0.069 .
LBM 7.081e-04 1.270e-03 0.557 0.591
Height -1.307e+00 8.298e-01 -1.575 0.150
---
Signif. codes: 0 ‘***’ 0.001 ‘**’ 0.01 ‘*’ 0.05 ‘.’ 0.1 ‘ ’ 1
Residual standard error: 12.74 on 9 degrees of freedom
Multiple R-squared: 0.2297,	Adjusted R-squared: 0.0585
F-statistic: 1.342 on 2 and 9 DF, p-value: 0.309
U16:
 Estimate Std. Error t value Pr(>|t|)
(Intercept) 1.350e+02 8.786e+01 1.536 0.133
LBM 7.759e-04 9.004e-04 0.862 0.395
Height -1.927e-01 6.078e-01 -0.317 0.753
Residual standard error: 19.85 on 36 degrees of freedom
 Multiple R-squared: 0.02157,	Adjusted R-squared: -0.03279
F-statistic: 0.3968 on 2 and 36 DF, p-value: 0.6754

## Slide 9
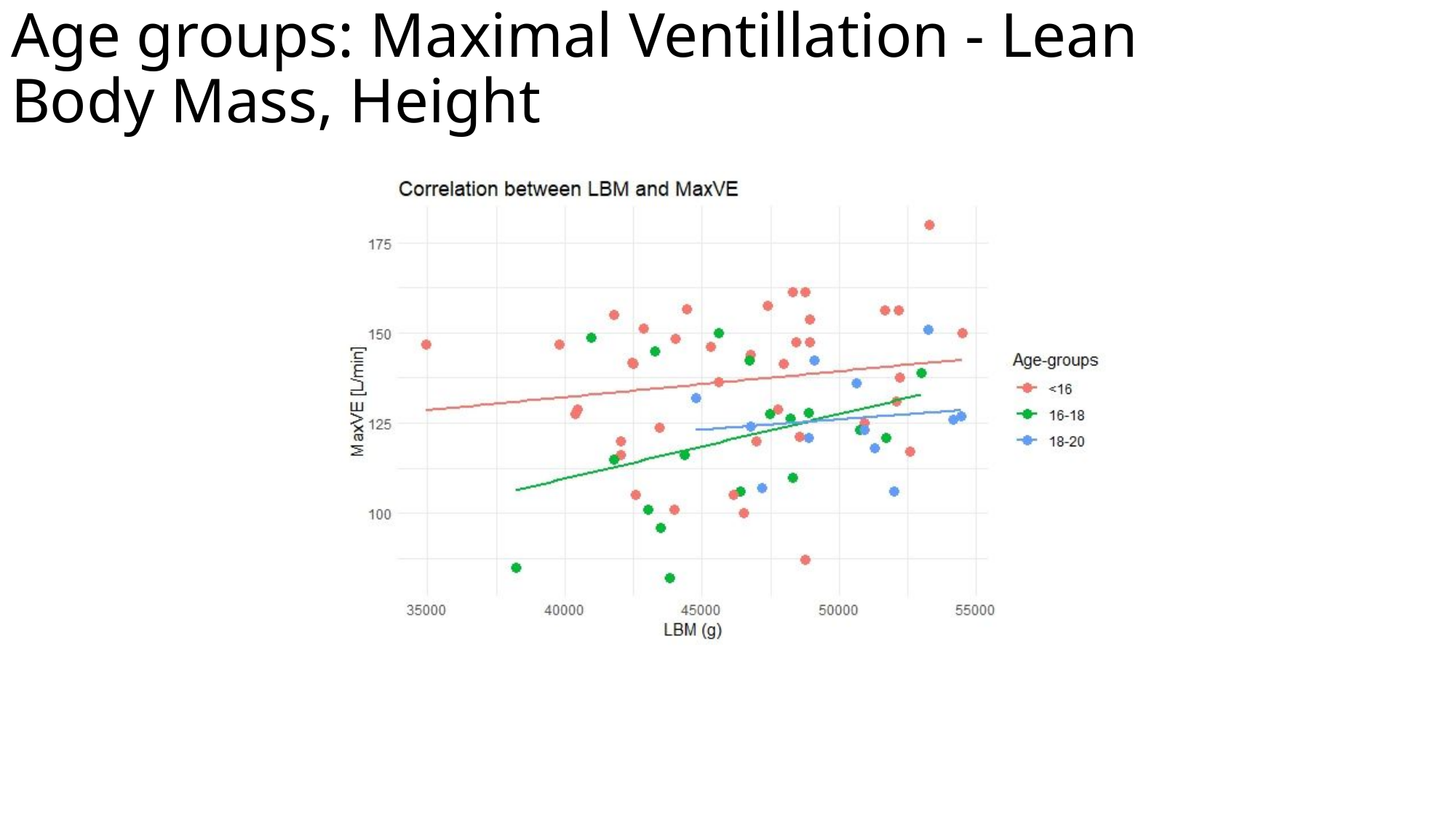

# Age groups: Maximal Ventillation - Lean Body Mass, Height

## Slide 10
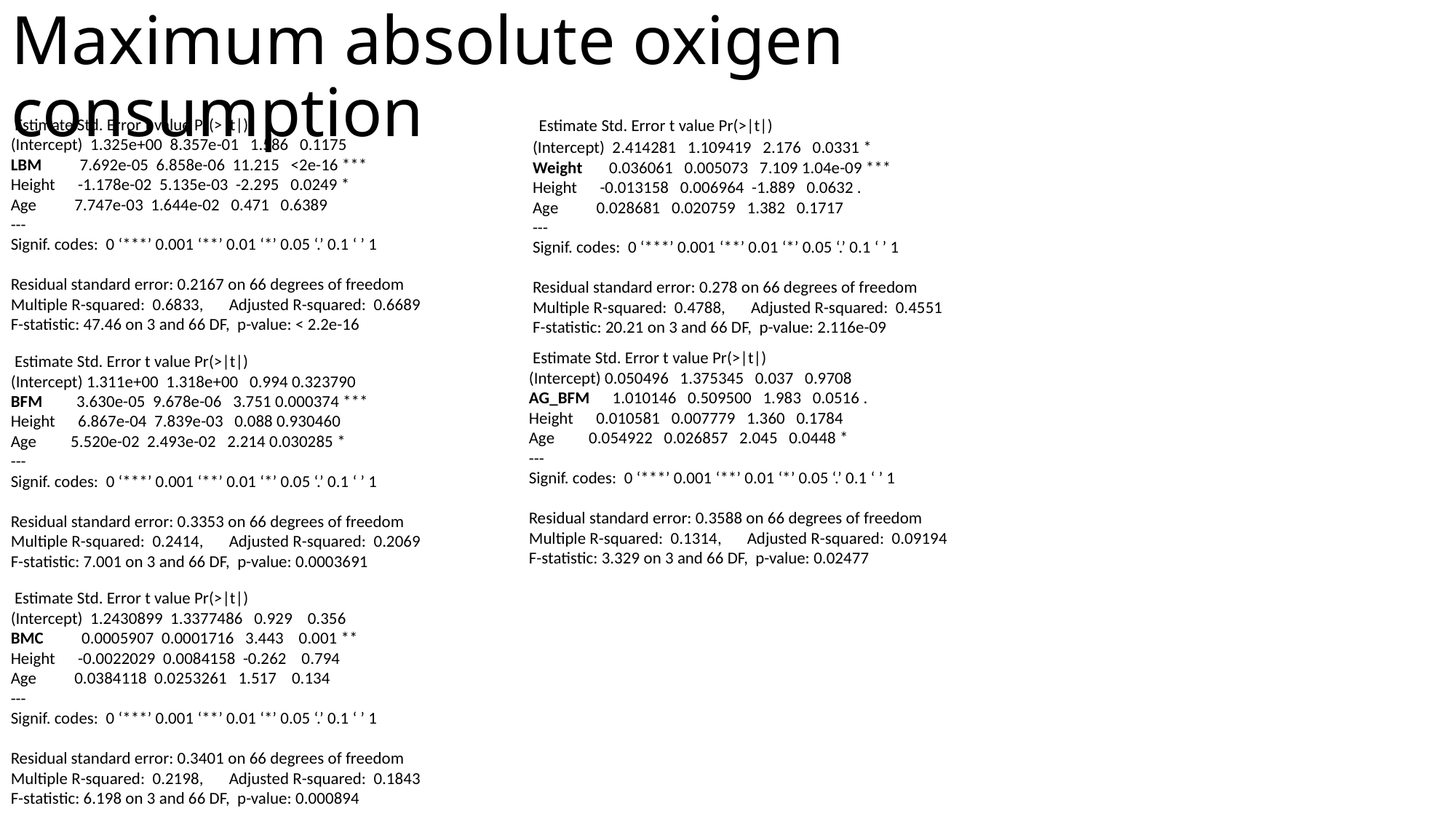

# Maximum absolute oxigen consumption
 Estimate Std. Error t value Pr(>|t|)
(Intercept) 2.414281 1.109419 2.176 0.0331 *
Weight 0.036061 0.005073 7.109 1.04e-09 ***
Height -0.013158 0.006964 -1.889 0.0632 .
Age 0.028681 0.020759 1.382 0.1717
---
Signif. codes: 0 ‘***’ 0.001 ‘**’ 0.01 ‘*’ 0.05 ‘.’ 0.1 ‘ ’ 1
Residual standard error: 0.278 on 66 degrees of freedom
Multiple R-squared: 0.4788,	Adjusted R-squared: 0.4551
F-statistic: 20.21 on 3 and 66 DF, p-value: 2.116e-09
 Estimate Std. Error t value Pr(>|t|)
(Intercept) 1.325e+00 8.357e-01 1.586 0.1175
LBM 7.692e-05 6.858e-06 11.215 <2e-16 ***
Height -1.178e-02 5.135e-03 -2.295 0.0249 *
Age 7.747e-03 1.644e-02 0.471 0.6389
---
Signif. codes: 0 ‘***’ 0.001 ‘**’ 0.01 ‘*’ 0.05 ‘.’ 0.1 ‘ ’ 1
Residual standard error: 0.2167 on 66 degrees of freedom
Multiple R-squared: 0.6833,	Adjusted R-squared: 0.6689
F-statistic: 47.46 on 3 and 66 DF, p-value: < 2.2e-16
 Estimate Std. Error t value Pr(>|t|)
(Intercept) 0.050496 1.375345 0.037 0.9708
AG_BFM 1.010146 0.509500 1.983 0.0516 .
Height 0.010581 0.007779 1.360 0.1784
Age 0.054922 0.026857 2.045 0.0448 *
---
Signif. codes: 0 ‘***’ 0.001 ‘**’ 0.01 ‘*’ 0.05 ‘.’ 0.1 ‘ ’ 1
Residual standard error: 0.3588 on 66 degrees of freedom
Multiple R-squared: 0.1314,	Adjusted R-squared: 0.09194
F-statistic: 3.329 on 3 and 66 DF, p-value: 0.02477
 Estimate Std. Error t value Pr(>|t|)
(Intercept) 1.311e+00 1.318e+00 0.994 0.323790
BFM 3.630e-05 9.678e-06 3.751 0.000374 ***
Height 6.867e-04 7.839e-03 0.088 0.930460
Age 5.520e-02 2.493e-02 2.214 0.030285 *
---
Signif. codes: 0 ‘***’ 0.001 ‘**’ 0.01 ‘*’ 0.05 ‘.’ 0.1 ‘ ’ 1
Residual standard error: 0.3353 on 66 degrees of freedom
Multiple R-squared: 0.2414,	Adjusted R-squared: 0.2069
F-statistic: 7.001 on 3 and 66 DF, p-value: 0.0003691
 Estimate Std. Error t value Pr(>|t|)
(Intercept) 1.2430899 1.3377486 0.929 0.356
BMC 0.0005907 0.0001716 3.443 0.001 **
Height -0.0022029 0.0084158 -0.262 0.794
Age 0.0384118 0.0253261 1.517 0.134
---
Signif. codes: 0 ‘***’ 0.001 ‘**’ 0.01 ‘*’ 0.05 ‘.’ 0.1 ‘ ’ 1
Residual standard error: 0.3401 on 66 degrees of freedom
Multiple R-squared: 0.2198,	Adjusted R-squared: 0.1843
F-statistic: 6.198 on 3 and 66 DF, p-value: 0.000894

## Slide 11
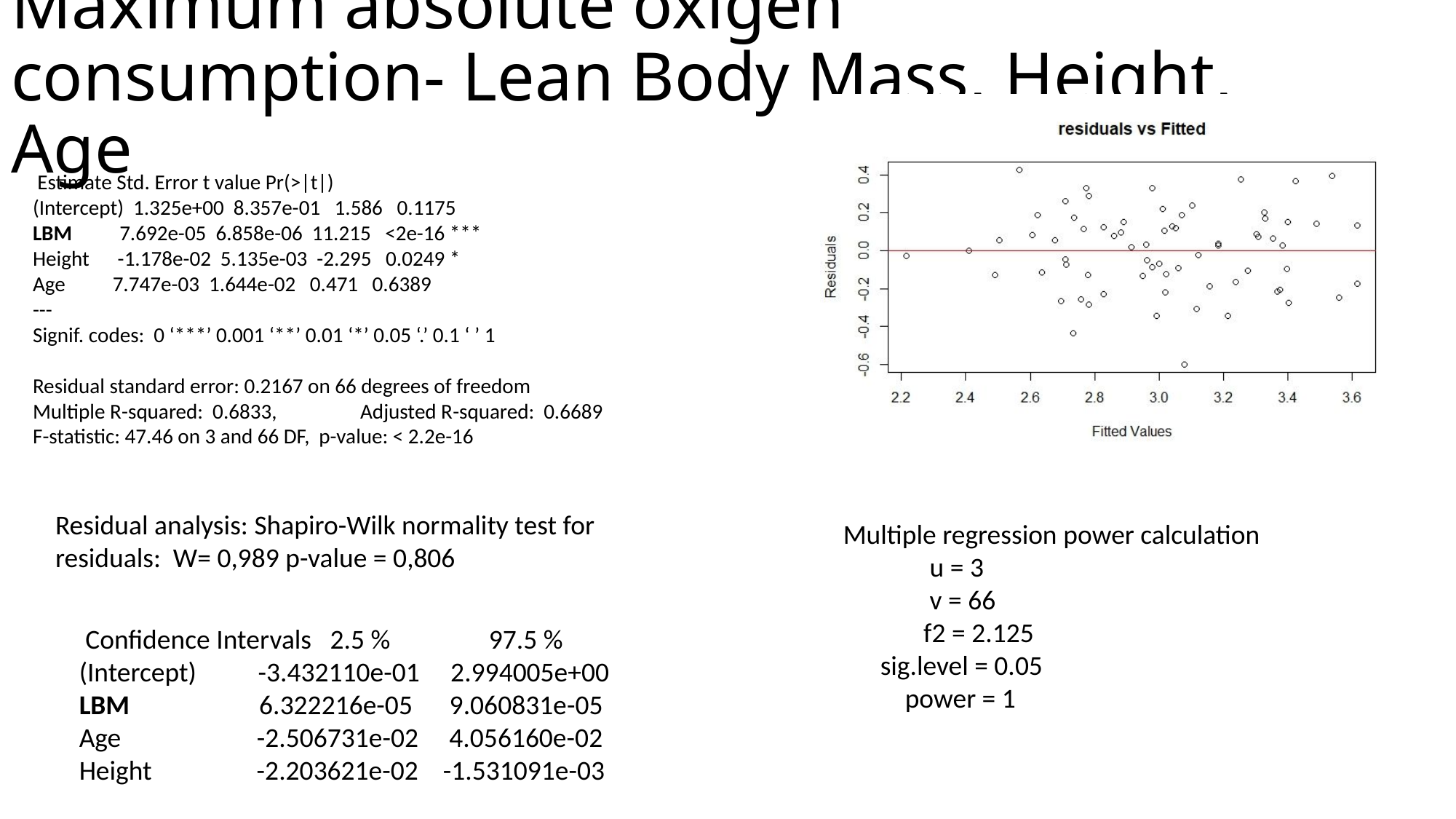

# Maximum absolute oxigen consumption- Lean Body Mass, Height, Age
 Estimate Std. Error t value Pr(>|t|)
(Intercept) 1.325e+00 8.357e-01 1.586 0.1175
LBM 7.692e-05 6.858e-06 11.215 <2e-16 ***
Height -1.178e-02 5.135e-03 -2.295 0.0249 *
Age 7.747e-03 1.644e-02 0.471 0.6389
---
Signif. codes: 0 ‘***’ 0.001 ‘**’ 0.01 ‘*’ 0.05 ‘.’ 0.1 ‘ ’ 1
Residual standard error: 0.2167 on 66 degrees of freedom
Multiple R-squared: 0.6833,	Adjusted R-squared: 0.6689
F-statistic: 47.46 on 3 and 66 DF, p-value: < 2.2e-16
Residual analysis: Shapiro-Wilk normality test for residuals: W= 0,989 p-value = 0,806
Multiple regression power calculation
 u = 3
 v = 66
 f2 = 2.125
 sig.level = 0.05
 power = 1
 Confidence Intervals 2.5 % 97.5 %
(Intercept) -3.432110e-01 2.994005e+00
LBM 6.322216e-05 9.060831e-05
Age -2.506731e-02 4.056160e-02
Height -2.203621e-02 -1.531091e-03

## Slide 12
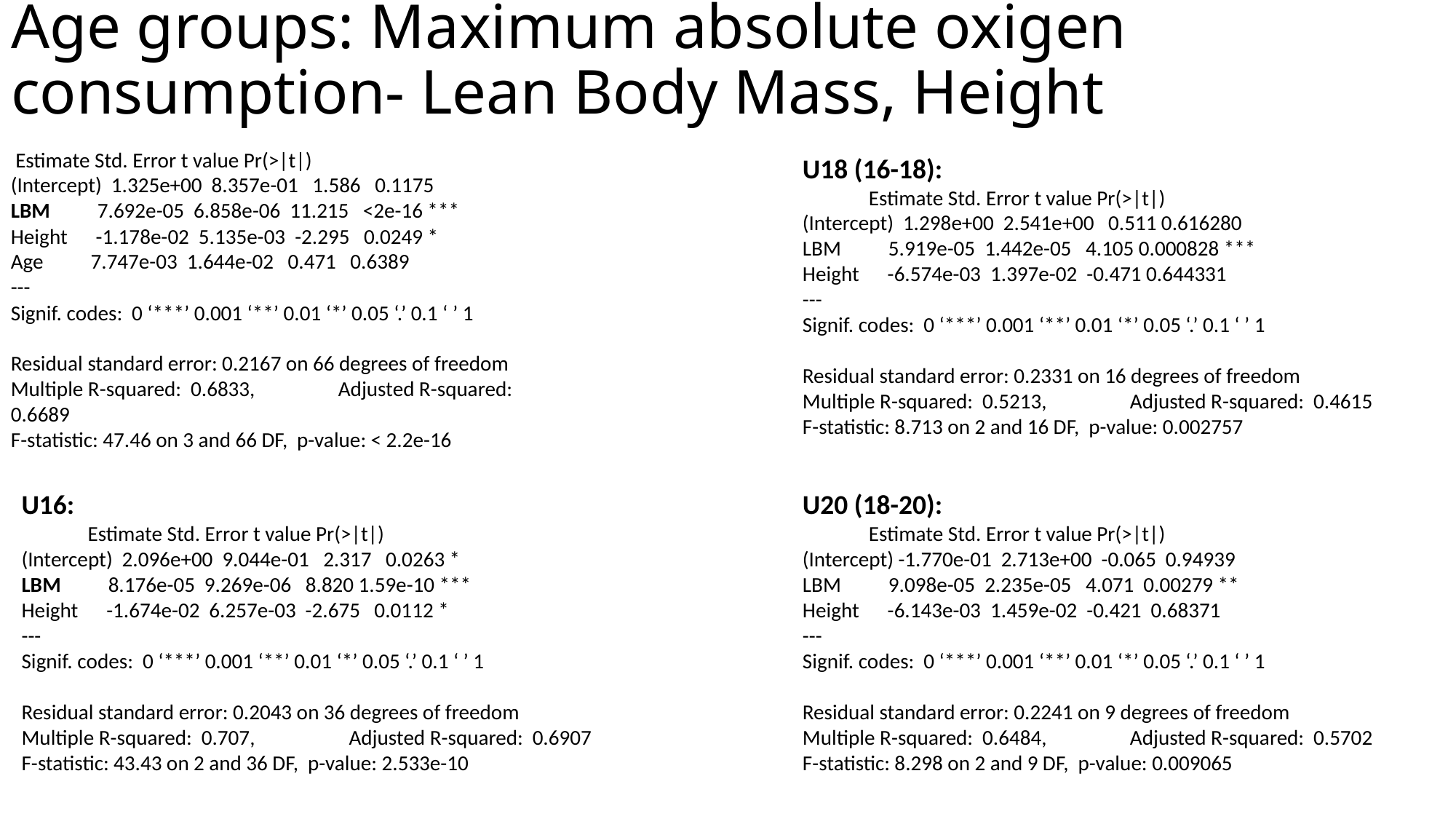

# Age groups: Maximum absolute oxigen consumption- Lean Body Mass, Height
 Estimate Std. Error t value Pr(>|t|)
(Intercept) 1.325e+00 8.357e-01 1.586 0.1175
LBM 7.692e-05 6.858e-06 11.215 <2e-16 ***
Height -1.178e-02 5.135e-03 -2.295 0.0249 *
Age 7.747e-03 1.644e-02 0.471 0.6389
---
Signif. codes: 0 ‘***’ 0.001 ‘**’ 0.01 ‘*’ 0.05 ‘.’ 0.1 ‘ ’ 1
Residual standard error: 0.2167 on 66 degrees of freedom
Multiple R-squared: 0.6833,	Adjusted R-squared: 0.6689
F-statistic: 47.46 on 3 and 66 DF, p-value: < 2.2e-16
U18 (16-18):
 Estimate Std. Error t value Pr(>|t|)
(Intercept) 1.298e+00 2.541e+00 0.511 0.616280
LBM 5.919e-05 1.442e-05 4.105 0.000828 ***
Height -6.574e-03 1.397e-02 -0.471 0.644331
---
Signif. codes: 0 ‘***’ 0.001 ‘**’ 0.01 ‘*’ 0.05 ‘.’ 0.1 ‘ ’ 1
Residual standard error: 0.2331 on 16 degrees of freedom
Multiple R-squared: 0.5213,	Adjusted R-squared: 0.4615
F-statistic: 8.713 on 2 and 16 DF, p-value: 0.002757
U16:
 Estimate Std. Error t value Pr(>|t|)
(Intercept) 2.096e+00 9.044e-01 2.317 0.0263 *
LBM 8.176e-05 9.269e-06 8.820 1.59e-10 ***
Height -1.674e-02 6.257e-03 -2.675 0.0112 *
---
Signif. codes: 0 ‘***’ 0.001 ‘**’ 0.01 ‘*’ 0.05 ‘.’ 0.1 ‘ ’ 1
Residual standard error: 0.2043 on 36 degrees of freedom
Multiple R-squared: 0.707,	Adjusted R-squared: 0.6907
F-statistic: 43.43 on 2 and 36 DF, p-value: 2.533e-10
U20 (18-20):
 Estimate Std. Error t value Pr(>|t|)
(Intercept) -1.770e-01 2.713e+00 -0.065 0.94939
LBM 9.098e-05 2.235e-05 4.071 0.00279 **
Height -6.143e-03 1.459e-02 -0.421 0.68371
---
Signif. codes: 0 ‘***’ 0.001 ‘**’ 0.01 ‘*’ 0.05 ‘.’ 0.1 ‘ ’ 1
Residual standard error: 0.2241 on 9 degrees of freedom
Multiple R-squared: 0.6484,	Adjusted R-squared: 0.5702
F-statistic: 8.298 on 2 and 9 DF, p-value: 0.009065

## Slide 13
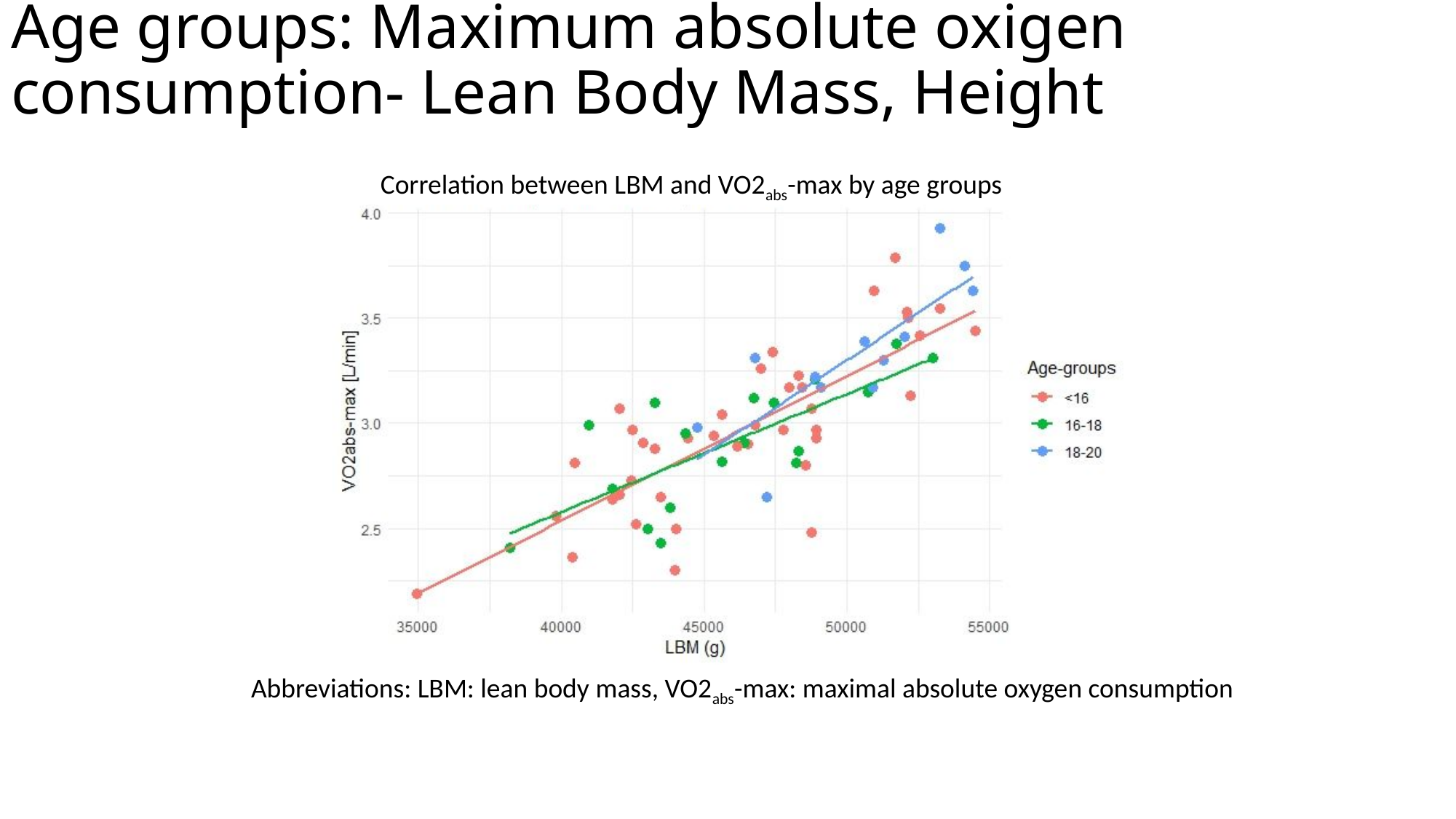

# Age groups: Maximum absolute oxigen consumption- Lean Body Mass, Height
Correlation between LBM and VO2abs-max by age groups
Abbreviations: LBM: lean body mass, VO2abs-max: maximal absolute oxygen consumption

## Slide 14
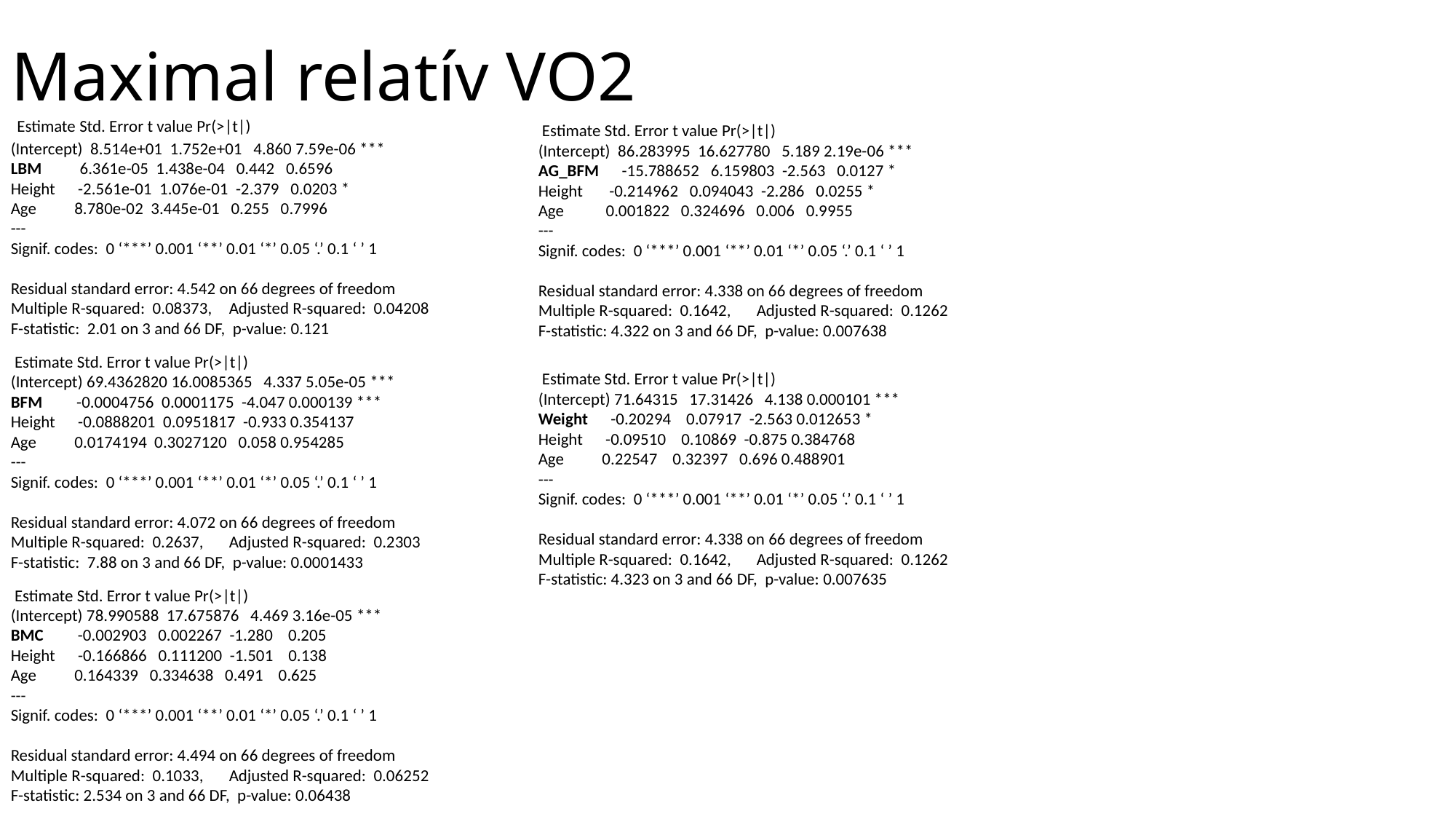

# Maximal relatív VO2
 Estimate Std. Error t value Pr(>|t|)
(Intercept) 8.514e+01 1.752e+01 4.860 7.59e-06 ***
LBM 6.361e-05 1.438e-04 0.442 0.6596
Height -2.561e-01 1.076e-01 -2.379 0.0203 *
Age 8.780e-02 3.445e-01 0.255 0.7996
---
Signif. codes: 0 ‘***’ 0.001 ‘**’ 0.01 ‘*’ 0.05 ‘.’ 0.1 ‘ ’ 1
Residual standard error: 4.542 on 66 degrees of freedom
Multiple R-squared: 0.08373,	Adjusted R-squared: 0.04208
F-statistic: 2.01 on 3 and 66 DF, p-value: 0.121
 Estimate Std. Error t value Pr(>|t|)
(Intercept) 86.283995 16.627780 5.189 2.19e-06 ***
AG_BFM -15.788652 6.159803 -2.563 0.0127 *
Height -0.214962 0.094043 -2.286 0.0255 *
Age 0.001822 0.324696 0.006 0.9955
---
Signif. codes: 0 ‘***’ 0.001 ‘**’ 0.01 ‘*’ 0.05 ‘.’ 0.1 ‘ ’ 1
Residual standard error: 4.338 on 66 degrees of freedom
Multiple R-squared: 0.1642,	Adjusted R-squared: 0.1262
F-statistic: 4.322 on 3 and 66 DF, p-value: 0.007638
 Estimate Std. Error t value Pr(>|t|)
(Intercept) 69.4362820 16.0085365 4.337 5.05e-05 ***
BFM -0.0004756 0.0001175 -4.047 0.000139 ***
Height -0.0888201 0.0951817 -0.933 0.354137
Age 0.0174194 0.3027120 0.058 0.954285
---
Signif. codes: 0 ‘***’ 0.001 ‘**’ 0.01 ‘*’ 0.05 ‘.’ 0.1 ‘ ’ 1
Residual standard error: 4.072 on 66 degrees of freedom
Multiple R-squared: 0.2637,	Adjusted R-squared: 0.2303
F-statistic: 7.88 on 3 and 66 DF, p-value: 0.0001433
 Estimate Std. Error t value Pr(>|t|)
(Intercept) 71.64315 17.31426 4.138 0.000101 ***
Weight -0.20294 0.07917 -2.563 0.012653 *
Height -0.09510 0.10869 -0.875 0.384768
Age 0.22547 0.32397 0.696 0.488901
---
Signif. codes: 0 ‘***’ 0.001 ‘**’ 0.01 ‘*’ 0.05 ‘.’ 0.1 ‘ ’ 1
Residual standard error: 4.338 on 66 degrees of freedom
Multiple R-squared: 0.1642,	Adjusted R-squared: 0.1262
F-statistic: 4.323 on 3 and 66 DF, p-value: 0.007635
 Estimate Std. Error t value Pr(>|t|)
(Intercept) 78.990588 17.675876 4.469 3.16e-05 ***
BMC -0.002903 0.002267 -1.280 0.205
Height -0.166866 0.111200 -1.501 0.138
Age 0.164339 0.334638 0.491 0.625
---
Signif. codes: 0 ‘***’ 0.001 ‘**’ 0.01 ‘*’ 0.05 ‘.’ 0.1 ‘ ’ 1
Residual standard error: 4.494 on 66 degrees of freedom
Multiple R-squared: 0.1033,	Adjusted R-squared: 0.06252
F-statistic: 2.534 on 3 and 66 DF, p-value: 0.06438

## Slide 15
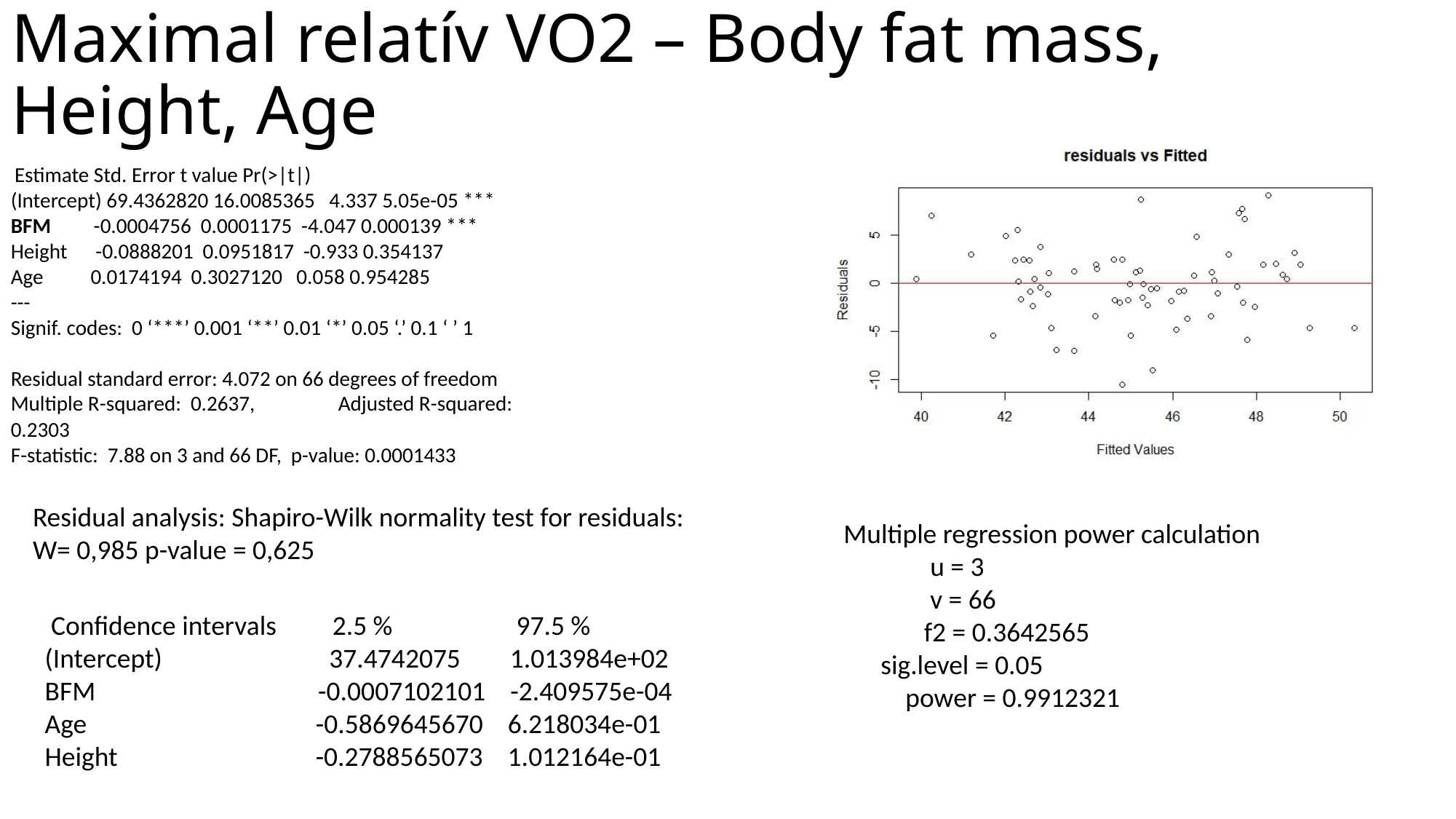

# Maximal relatív VO2 – Body fat mass, Height, Age
 Estimate Std. Error t value Pr(>|t|)
(Intercept) 69.4362820 16.0085365 4.337 5.05e-05 ***
BFM -0.0004756 0.0001175 -4.047 0.000139 ***
Height -0.0888201 0.0951817 -0.933 0.354137
Age 0.0174194 0.3027120 0.058 0.954285
---
Signif. codes: 0 ‘***’ 0.001 ‘**’ 0.01 ‘*’ 0.05 ‘.’ 0.1 ‘ ’ 1
Residual standard error: 4.072 on 66 degrees of freedom
Multiple R-squared: 0.2637,	Adjusted R-squared: 0.2303
F-statistic: 7.88 on 3 and 66 DF, p-value: 0.0001433
Residual analysis: Shapiro-Wilk normality test for residuals:
W= 0,985 p-value = 0,625
Multiple regression power calculation
 u = 3
 v = 66
 f2 = 0.3642565
 sig.level = 0.05
 power = 0.9912321
 Confidence intervals 2.5 % 97.5 %
(Intercept) 37.4742075 1.013984e+02
BFM -0.0007102101 -2.409575e-04
Age -0.5869645670 6.218034e-01
Height -0.2788565073 1.012164e-01

## Slide 16
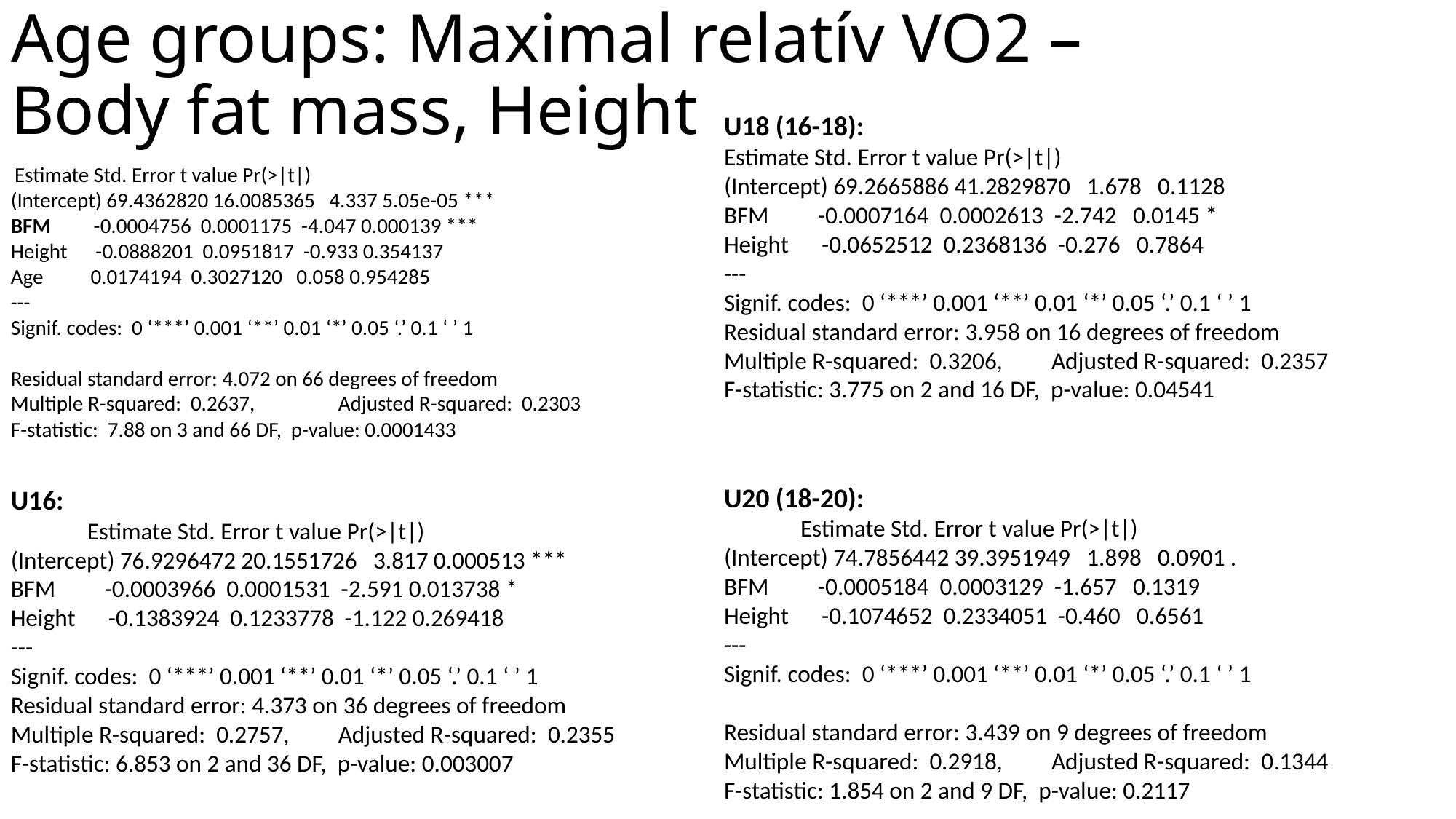

# Age groups: Maximal relatív VO2 – Body fat mass, Height
U18 (16-18):
Estimate Std. Error t value Pr(>|t|)
(Intercept) 69.2665886 41.2829870 1.678 0.1128
BFM -0.0007164 0.0002613 -2.742 0.0145 *
Height -0.0652512 0.2368136 -0.276 0.7864
---
Signif. codes: 0 ‘***’ 0.001 ‘**’ 0.01 ‘*’ 0.05 ‘.’ 0.1 ‘ ’ 1
Residual standard error: 3.958 on 16 degrees of freedom
Multiple R-squared: 0.3206,	Adjusted R-squared: 0.2357
F-statistic: 3.775 on 2 and 16 DF, p-value: 0.04541
 Estimate Std. Error t value Pr(>|t|)
(Intercept) 69.4362820 16.0085365 4.337 5.05e-05 ***
BFM -0.0004756 0.0001175 -4.047 0.000139 ***
Height -0.0888201 0.0951817 -0.933 0.354137
Age 0.0174194 0.3027120 0.058 0.954285
---
Signif. codes: 0 ‘***’ 0.001 ‘**’ 0.01 ‘*’ 0.05 ‘.’ 0.1 ‘ ’ 1
Residual standard error: 4.072 on 66 degrees of freedom
Multiple R-squared: 0.2637,	Adjusted R-squared: 0.2303
F-statistic: 7.88 on 3 and 66 DF, p-value: 0.0001433
U20 (18-20):
 Estimate Std. Error t value Pr(>|t|)
(Intercept) 74.7856442 39.3951949 1.898 0.0901 .
BFM -0.0005184 0.0003129 -1.657 0.1319
Height -0.1074652 0.2334051 -0.460 0.6561
---
Signif. codes: 0 ‘***’ 0.001 ‘**’ 0.01 ‘*’ 0.05 ‘.’ 0.1 ‘ ’ 1
Residual standard error: 3.439 on 9 degrees of freedom
Multiple R-squared: 0.2918,	Adjusted R-squared: 0.1344
F-statistic: 1.854 on 2 and 9 DF, p-value: 0.2117
U16:
 Estimate Std. Error t value Pr(>|t|)
(Intercept) 76.9296472 20.1551726 3.817 0.000513 ***
BFM -0.0003966 0.0001531 -2.591 0.013738 *
Height -0.1383924 0.1233778 -1.122 0.269418
---
Signif. codes: 0 ‘***’ 0.001 ‘**’ 0.01 ‘*’ 0.05 ‘.’ 0.1 ‘ ’ 1
Residual standard error: 4.373 on 36 degrees of freedom
Multiple R-squared: 0.2757,	Adjusted R-squared: 0.2355
F-statistic: 6.853 on 2 and 36 DF, p-value: 0.003007

## Slide 17
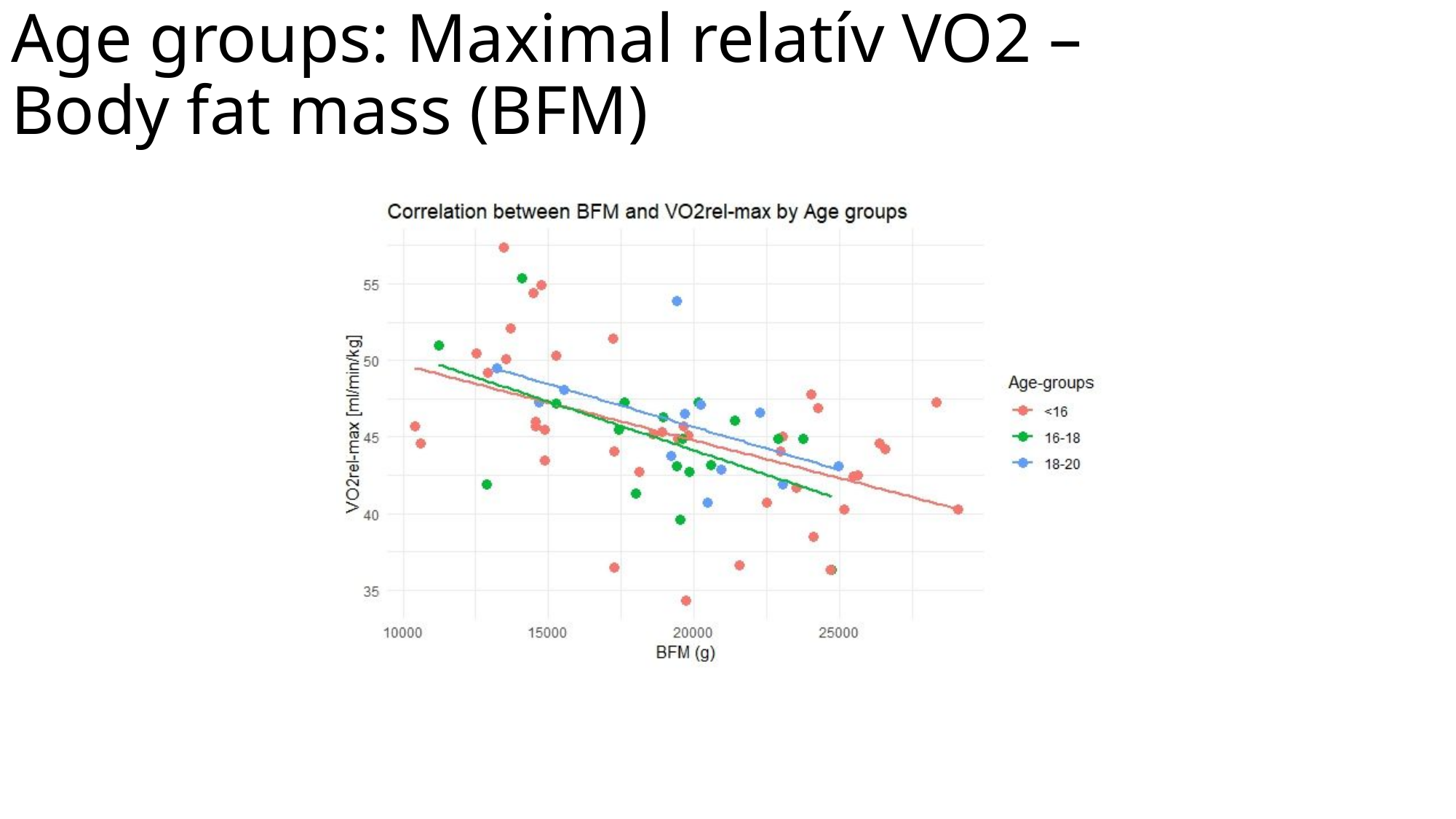

# Age groups: Maximal relatív VO2 – Body fat mass (BFM)

## Slide 18
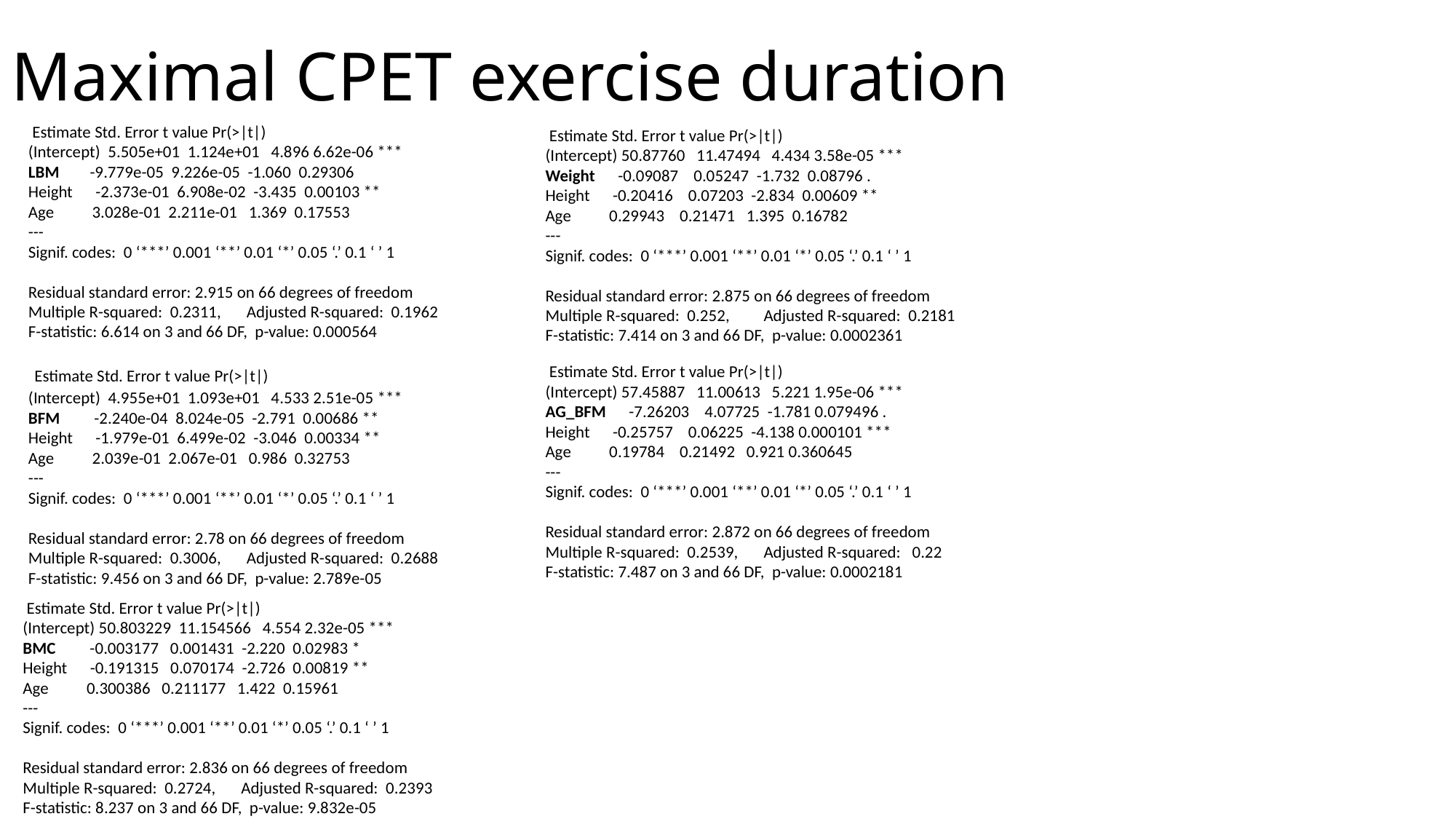

# Maximal CPET exercise duration
 Estimate Std. Error t value Pr(>|t|)
(Intercept) 5.505e+01 1.124e+01 4.896 6.62e-06 ***
LBM -9.779e-05 9.226e-05 -1.060 0.29306
Height -2.373e-01 6.908e-02 -3.435 0.00103 **
Age 3.028e-01 2.211e-01 1.369 0.17553
---
Signif. codes: 0 ‘***’ 0.001 ‘**’ 0.01 ‘*’ 0.05 ‘.’ 0.1 ‘ ’ 1
Residual standard error: 2.915 on 66 degrees of freedom
Multiple R-squared: 0.2311,	Adjusted R-squared: 0.1962
F-statistic: 6.614 on 3 and 66 DF, p-value: 0.000564
 Estimate Std. Error t value Pr(>|t|)
(Intercept) 50.87760 11.47494 4.434 3.58e-05 ***
Weight -0.09087 0.05247 -1.732 0.08796 .
Height -0.20416 0.07203 -2.834 0.00609 **
Age 0.29943 0.21471 1.395 0.16782
---
Signif. codes: 0 ‘***’ 0.001 ‘**’ 0.01 ‘*’ 0.05 ‘.’ 0.1 ‘ ’ 1
Residual standard error: 2.875 on 66 degrees of freedom
Multiple R-squared: 0.252,	Adjusted R-squared: 0.2181
F-statistic: 7.414 on 3 and 66 DF, p-value: 0.0002361
 Estimate Std. Error t value Pr(>|t|)
(Intercept) 4.955e+01 1.093e+01 4.533 2.51e-05 ***
BFM -2.240e-04 8.024e-05 -2.791 0.00686 **
Height -1.979e-01 6.499e-02 -3.046 0.00334 **
Age 2.039e-01 2.067e-01 0.986 0.32753
---
Signif. codes: 0 ‘***’ 0.001 ‘**’ 0.01 ‘*’ 0.05 ‘.’ 0.1 ‘ ’ 1
Residual standard error: 2.78 on 66 degrees of freedom
Multiple R-squared: 0.3006,	Adjusted R-squared: 0.2688
F-statistic: 9.456 on 3 and 66 DF, p-value: 2.789e-05
 Estimate Std. Error t value Pr(>|t|)
(Intercept) 57.45887 11.00613 5.221 1.95e-06 ***
AG_BFM -7.26203 4.07725 -1.781 0.079496 .
Height -0.25757 0.06225 -4.138 0.000101 ***
Age 0.19784 0.21492 0.921 0.360645
---
Signif. codes: 0 ‘***’ 0.001 ‘**’ 0.01 ‘*’ 0.05 ‘.’ 0.1 ‘ ’ 1
Residual standard error: 2.872 on 66 degrees of freedom
Multiple R-squared: 0.2539,	Adjusted R-squared: 0.22
F-statistic: 7.487 on 3 and 66 DF, p-value: 0.0002181
 Estimate Std. Error t value Pr(>|t|)
(Intercept) 50.803229 11.154566 4.554 2.32e-05 ***
BMC -0.003177 0.001431 -2.220 0.02983 *
Height -0.191315 0.070174 -2.726 0.00819 **
Age 0.300386 0.211177 1.422 0.15961
---
Signif. codes: 0 ‘***’ 0.001 ‘**’ 0.01 ‘*’ 0.05 ‘.’ 0.1 ‘ ’ 1
Residual standard error: 2.836 on 66 degrees of freedom
Multiple R-squared: 0.2724,	Adjusted R-squared: 0.2393
F-statistic: 8.237 on 3 and 66 DF, p-value: 9.832e-05

## Slide 19
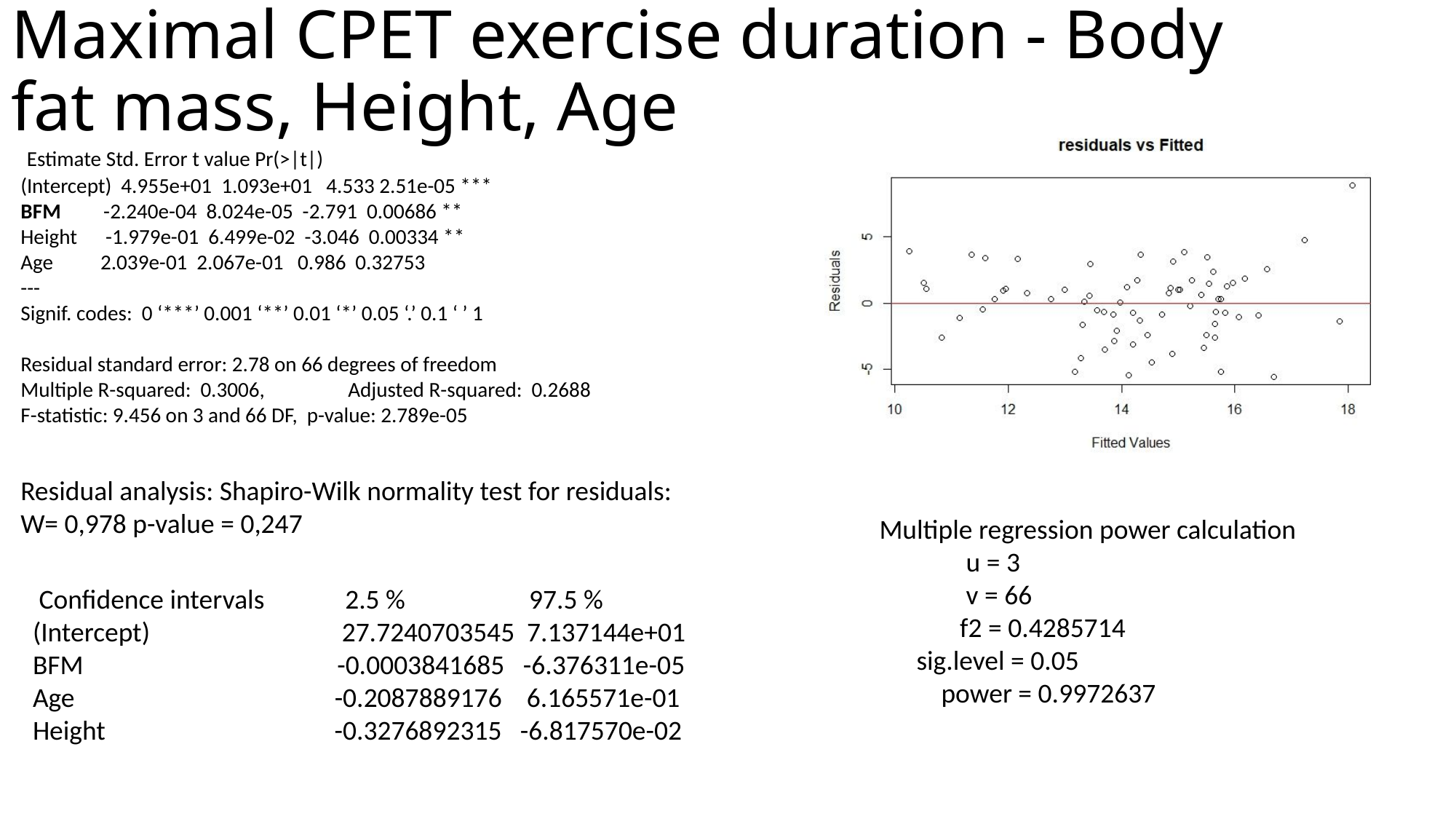

# Maximal CPET exercise duration - Body fat mass, Height, Age
 Estimate Std. Error t value Pr(>|t|)
(Intercept) 4.955e+01 1.093e+01 4.533 2.51e-05 ***
BFM -2.240e-04 8.024e-05 -2.791 0.00686 **
Height -1.979e-01 6.499e-02 -3.046 0.00334 **
Age 2.039e-01 2.067e-01 0.986 0.32753
---
Signif. codes: 0 ‘***’ 0.001 ‘**’ 0.01 ‘*’ 0.05 ‘.’ 0.1 ‘ ’ 1
Residual standard error: 2.78 on 66 degrees of freedom
Multiple R-squared: 0.3006,	Adjusted R-squared: 0.2688
F-statistic: 9.456 on 3 and 66 DF, p-value: 2.789e-05
Residual analysis: Shapiro-Wilk normality test for residuals:
W= 0,978 p-value = 0,247
Multiple regression power calculation
 u = 3
 v = 66
 f2 = 0.4285714
 sig.level = 0.05
 power = 0.9972637
 Confidence intervals 2.5 % 97.5 %
(Intercept) 27.7240703545 7.137144e+01
BFM -0.0003841685 -6.376311e-05
Age -0.2087889176 6.165571e-01
Height -0.3276892315 -6.817570e-02

## Slide 20
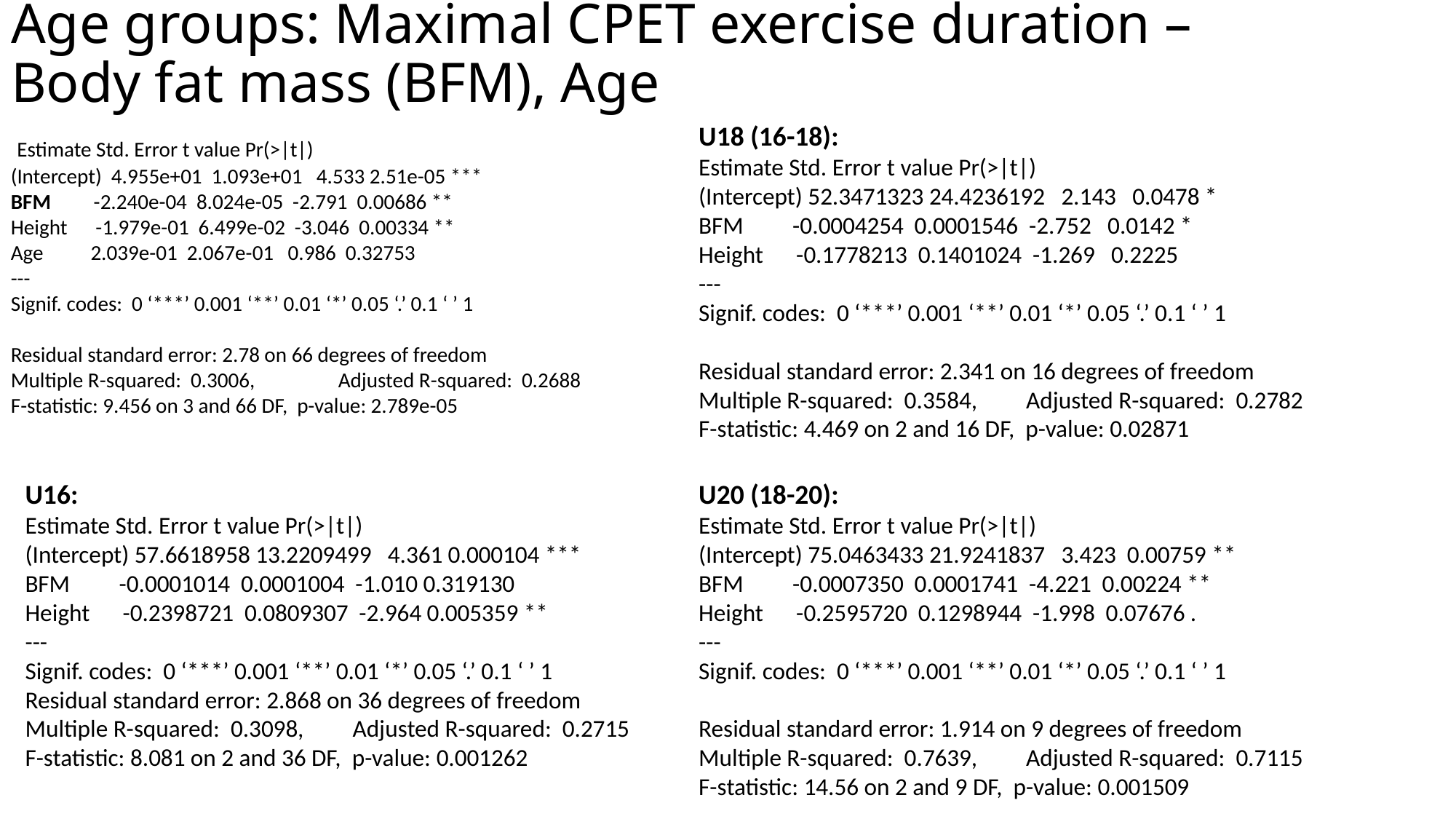

# Age groups: Maximal CPET exercise duration – Body fat mass (BFM), Age
U18 (16-18):
Estimate Std. Error t value Pr(>|t|)
(Intercept) 52.3471323 24.4236192 2.143 0.0478 *
BFM -0.0004254 0.0001546 -2.752 0.0142 *
Height -0.1778213 0.1401024 -1.269 0.2225
---
Signif. codes: 0 ‘***’ 0.001 ‘**’ 0.01 ‘*’ 0.05 ‘.’ 0.1 ‘ ’ 1
Residual standard error: 2.341 on 16 degrees of freedom
Multiple R-squared: 0.3584,	Adjusted R-squared: 0.2782
F-statistic: 4.469 on 2 and 16 DF, p-value: 0.02871
 Estimate Std. Error t value Pr(>|t|)
(Intercept) 4.955e+01 1.093e+01 4.533 2.51e-05 ***
BFM -2.240e-04 8.024e-05 -2.791 0.00686 **
Height -1.979e-01 6.499e-02 -3.046 0.00334 **
Age 2.039e-01 2.067e-01 0.986 0.32753
---
Signif. codes: 0 ‘***’ 0.001 ‘**’ 0.01 ‘*’ 0.05 ‘.’ 0.1 ‘ ’ 1
Residual standard error: 2.78 on 66 degrees of freedom
Multiple R-squared: 0.3006,	Adjusted R-squared: 0.2688
F-statistic: 9.456 on 3 and 66 DF, p-value: 2.789e-05
U16:
Estimate Std. Error t value Pr(>|t|)
(Intercept) 57.6618958 13.2209499 4.361 0.000104 ***
BFM -0.0001014 0.0001004 -1.010 0.319130
Height -0.2398721 0.0809307 -2.964 0.005359 **
---
Signif. codes: 0 ‘***’ 0.001 ‘**’ 0.01 ‘*’ 0.05 ‘.’ 0.1 ‘ ’ 1
Residual standard error: 2.868 on 36 degrees of freedom
Multiple R-squared: 0.3098,	Adjusted R-squared: 0.2715
F-statistic: 8.081 on 2 and 36 DF, p-value: 0.001262
U20 (18-20):
Estimate Std. Error t value Pr(>|t|)
(Intercept) 75.0463433 21.9241837 3.423 0.00759 **
BFM -0.0007350 0.0001741 -4.221 0.00224 **
Height -0.2595720 0.1298944 -1.998 0.07676 .
---
Signif. codes: 0 ‘***’ 0.001 ‘**’ 0.01 ‘*’ 0.05 ‘.’ 0.1 ‘ ’ 1
Residual standard error: 1.914 on 9 degrees of freedom
Multiple R-squared: 0.7639,	Adjusted R-squared: 0.7115
F-statistic: 14.56 on 2 and 9 DF, p-value: 0.001509

## Slide 21
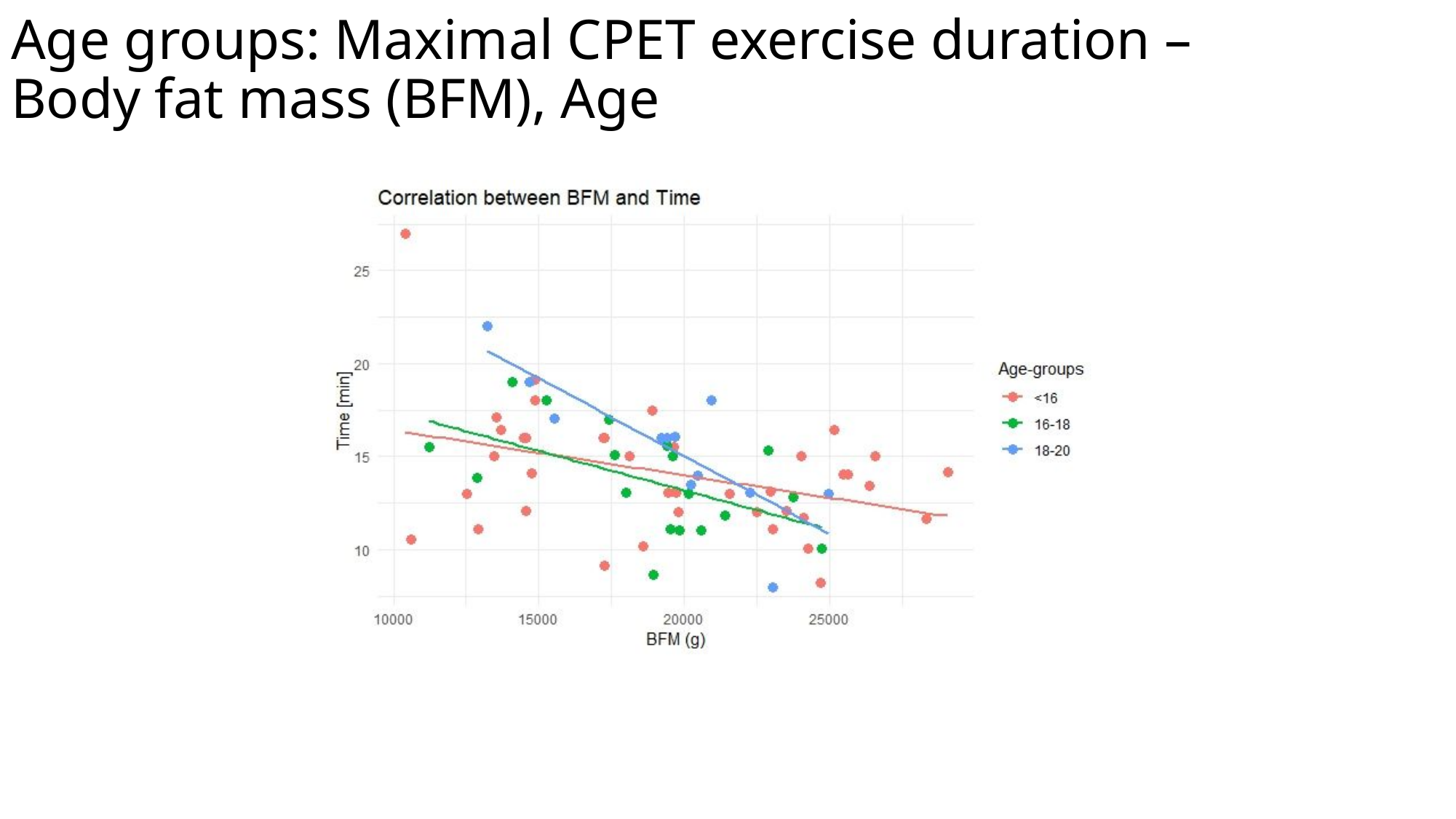

# Age groups: Maximal CPET exercise duration – Body fat mass (BFM), Age

## Slide 22
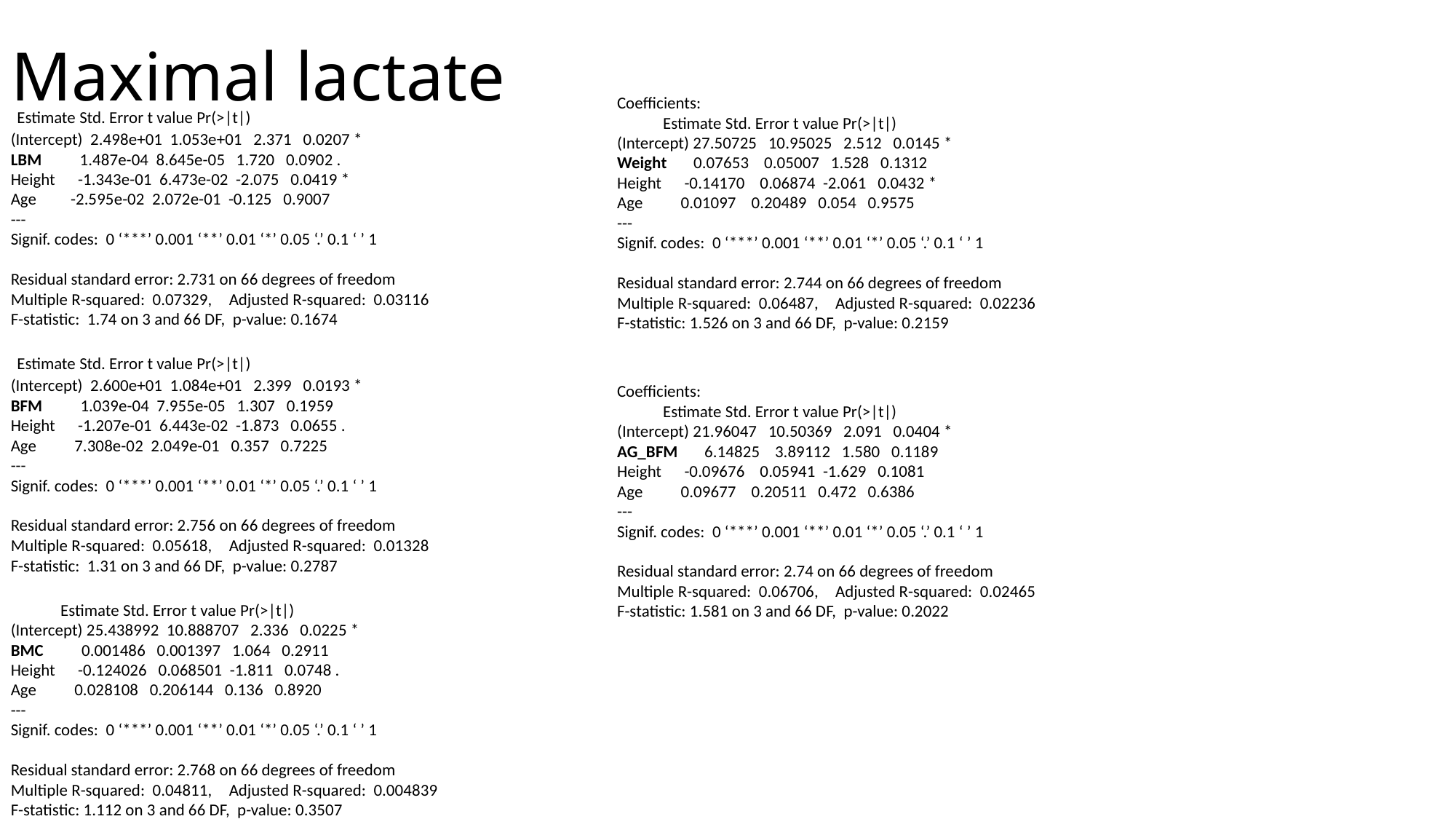

# Maximal lactate
Coefficients:
 Estimate Std. Error t value Pr(>|t|)
(Intercept) 27.50725 10.95025 2.512 0.0145 *
Weight 0.07653 0.05007 1.528 0.1312
Height -0.14170 0.06874 -2.061 0.0432 *
Age 0.01097 0.20489 0.054 0.9575
---
Signif. codes: 0 ‘***’ 0.001 ‘**’ 0.01 ‘*’ 0.05 ‘.’ 0.1 ‘ ’ 1
Residual standard error: 2.744 on 66 degrees of freedom
Multiple R-squared: 0.06487,	Adjusted R-squared: 0.02236
F-statistic: 1.526 on 3 and 66 DF, p-value: 0.2159
 Estimate Std. Error t value Pr(>|t|)
(Intercept) 2.498e+01 1.053e+01 2.371 0.0207 *
LBM 1.487e-04 8.645e-05 1.720 0.0902 .
Height -1.343e-01 6.473e-02 -2.075 0.0419 *
Age -2.595e-02 2.072e-01 -0.125 0.9007
---
Signif. codes: 0 ‘***’ 0.001 ‘**’ 0.01 ‘*’ 0.05 ‘.’ 0.1 ‘ ’ 1
Residual standard error: 2.731 on 66 degrees of freedom
Multiple R-squared: 0.07329,	Adjusted R-squared: 0.03116
F-statistic: 1.74 on 3 and 66 DF, p-value: 0.1674
 Estimate Std. Error t value Pr(>|t|)
(Intercept) 2.600e+01 1.084e+01 2.399 0.0193 *
BFM 1.039e-04 7.955e-05 1.307 0.1959
Height -1.207e-01 6.443e-02 -1.873 0.0655 .
Age 7.308e-02 2.049e-01 0.357 0.7225
---
Signif. codes: 0 ‘***’ 0.001 ‘**’ 0.01 ‘*’ 0.05 ‘.’ 0.1 ‘ ’ 1
Residual standard error: 2.756 on 66 degrees of freedom
Multiple R-squared: 0.05618,	Adjusted R-squared: 0.01328
F-statistic: 1.31 on 3 and 66 DF, p-value: 0.2787
Coefficients:
 Estimate Std. Error t value Pr(>|t|)
(Intercept) 21.96047 10.50369 2.091 0.0404 *
AG_BFM 6.14825 3.89112 1.580 0.1189
Height -0.09676 0.05941 -1.629 0.1081
Age 0.09677 0.20511 0.472 0.6386
---
Signif. codes: 0 ‘***’ 0.001 ‘**’ 0.01 ‘*’ 0.05 ‘.’ 0.1 ‘ ’ 1
Residual standard error: 2.74 on 66 degrees of freedom
Multiple R-squared: 0.06706,	Adjusted R-squared: 0.02465
F-statistic: 1.581 on 3 and 66 DF, p-value: 0.2022
 Estimate Std. Error t value Pr(>|t|)
(Intercept) 25.438992 10.888707 2.336 0.0225 *
BMC 0.001486 0.001397 1.064 0.2911
Height -0.124026 0.068501 -1.811 0.0748 .
Age 0.028108 0.206144 0.136 0.8920
---
Signif. codes: 0 ‘***’ 0.001 ‘**’ 0.01 ‘*’ 0.05 ‘.’ 0.1 ‘ ’ 1
Residual standard error: 2.768 on 66 degrees of freedom
Multiple R-squared: 0.04811,	Adjusted R-squared: 0.004839
F-statistic: 1.112 on 3 and 66 DF, p-value: 0.3507

## Slide 23
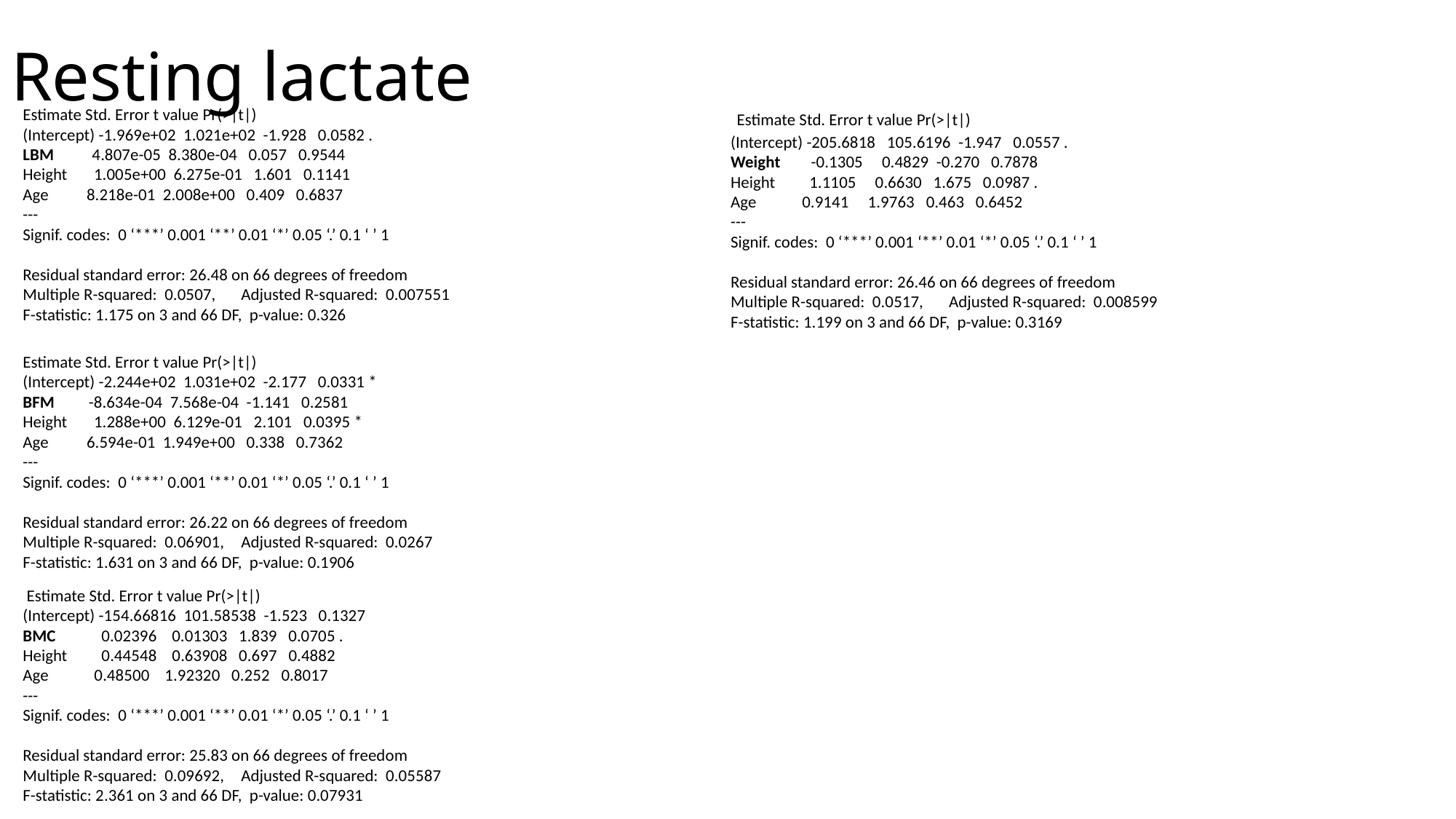

# Resting lactate
 Estimate Std. Error t value Pr(>|t|)
(Intercept) -205.6818 105.6196 -1.947 0.0557 .
Weight -0.1305 0.4829 -0.270 0.7878
Height 1.1105 0.6630 1.675 0.0987 .
Age 0.9141 1.9763 0.463 0.6452
---
Signif. codes: 0 ‘***’ 0.001 ‘**’ 0.01 ‘*’ 0.05 ‘.’ 0.1 ‘ ’ 1
Residual standard error: 26.46 on 66 degrees of freedom
Multiple R-squared: 0.0517,	Adjusted R-squared: 0.008599
F-statistic: 1.199 on 3 and 66 DF, p-value: 0.3169
Estimate Std. Error t value Pr(>|t|)
(Intercept) -1.969e+02 1.021e+02 -1.928 0.0582 .
LBM 4.807e-05 8.380e-04 0.057 0.9544
Height 1.005e+00 6.275e-01 1.601 0.1141
Age 8.218e-01 2.008e+00 0.409 0.6837
---
Signif. codes: 0 ‘***’ 0.001 ‘**’ 0.01 ‘*’ 0.05 ‘.’ 0.1 ‘ ’ 1
Residual standard error: 26.48 on 66 degrees of freedom
Multiple R-squared: 0.0507,	Adjusted R-squared: 0.007551
F-statistic: 1.175 on 3 and 66 DF, p-value: 0.326
Estimate Std. Error t value Pr(>|t|)
(Intercept) -2.244e+02 1.031e+02 -2.177 0.0331 *
BFM -8.634e-04 7.568e-04 -1.141 0.2581
Height 1.288e+00 6.129e-01 2.101 0.0395 *
Age 6.594e-01 1.949e+00 0.338 0.7362
---
Signif. codes: 0 ‘***’ 0.001 ‘**’ 0.01 ‘*’ 0.05 ‘.’ 0.1 ‘ ’ 1
Residual standard error: 26.22 on 66 degrees of freedom
Multiple R-squared: 0.06901,	Adjusted R-squared: 0.0267
F-statistic: 1.631 on 3 and 66 DF, p-value: 0.1906
 Estimate Std. Error t value Pr(>|t|)
(Intercept) -154.66816 101.58538 -1.523 0.1327
BMC 0.02396 0.01303 1.839 0.0705 .
Height 0.44548 0.63908 0.697 0.4882
Age 0.48500 1.92320 0.252 0.8017
---
Signif. codes: 0 ‘***’ 0.001 ‘**’ 0.01 ‘*’ 0.05 ‘.’ 0.1 ‘ ’ 1
Residual standard error: 25.83 on 66 degrees of freedom
Multiple R-squared: 0.09692,	Adjusted R-squared: 0.05587
F-statistic: 2.361 on 3 and 66 DF, p-value: 0.07931
